# Genomic and Genetic Diversity within the *Pseudomonas fluorescens* Complex

**DOI:** 10.1371/journal.pone.0150183

**Published:** 2016-02-25

**Authors:** Daniel Garrido-Sanz, Jan P. Meier-Kolthoff, Markus Göker, Marta Martín, Rafael Rivilla, Miguel Redondo-Nieto

**Affiliations:** 1 Departamento de Biología, Facultad de Ciencias, Universidad Autónoma de Madrid, c/Darwin, 2, Madrid, 28049, Spain; 2 Leibniz Institute DSMZ–German Collection of Microorganisms and Cell Cultures, Inhoffenstraße 7B, 38124, Braunschweig, Germany; Virginia Tech, UNITED STATES

## Abstract

The *Pseudomonas fluorescens* complex includes *Pseudomonas* strains that have been taxonomically assigned to more than fifty different species, many of which have been described as plant growth-promoting rhizobacteria (PGPR) with potential applications in biocontrol and biofertilization. So far the phylogeny of this complex has been analyzed according to phenotypic traits, 16S rDNA, MLSA and inferred by whole-genome analysis. However, since most of the type strains have not been fully sequenced and new species are frequently described, correlation between taxonomy and phylogenomic analysis is missing. In recent years, the genomes of a large number of strains have been sequenced, showing important genomic heterogeneity and providing information suitable for genomic studies that are important to understand the genomic and genetic diversity shown by strains of this complex. Based on MLSA and several whole-genome sequence-based analyses of 93 sequenced strains, we have divided the *P*. *fluorescens* complex into eight phylogenomic groups that agree with previous works based on type strains. Digital DDH (dDDH) identified 69 species and 75 subspecies within the 93 genomes. The eight groups corresponded to clustering with a threshold of 31.8% dDDH, in full agreement with our MLSA. The Average Nucleotide Identity (ANI) approach showed inconsistencies regarding the assignment to species and to the eight groups. The small core genome of 1,334 CDSs and the large pan-genome of 30,848 CDSs, show the large diversity and genetic heterogeneity of the *P*. *fluorescens* complex. However, a low number of strains were enough to explain most of the CDSs diversity at core and strain-specific genomic fractions. Finally, the identification and analysis of group-specific genome and the screening for distinctive characters revealed a phylogenomic distribution of traits among the groups that provided insights into biocontrol and bioremediation applications as well as their role as PGPR.

## Introduction

Pseudomonads are gram-negative γ-proteobacteria widely distributed in a variety of environments and well known for their metabolic versatility in the utilization of organic compounds as energy and carbon sources [1, 2] and production of diverse secondary metabolites [3, 4]. *Pseudomonas* is one of the most complex and diverse genera, which is reflected in the more than 100 species described to date [5] and since first being described [6], the taxonomical has suffered many changes. Several studies have informally assessed the presence of groups and subgroups within the genera [7, 8], one of which is the *Pseudomonas fluorescens* group, where more than 50 validly named species have been described and it has been divided into subgroups that differ from multilocus sequence analysis (MLSA) and phylogenomic analysis [7–10]. The *P*. *fluorescens* group contains several species (*P*. *brassicacearum*, *P*. *protegens*, *P*. *chlororaphis*, and *P*. *fluorescens*) described as PGPR due to their ability to suppress plant diseases caused by pathogens [4, 11, 12] via competitive colonization of plant tissues [13], production of antibiotics [14–16], induction of systemic resistance responses in the plant [17, 18], and production of phytohormones or metabolites that modify the plant’s hormonal balance [19]. All these features make these strains particularly suitable for biocontrol and biofertilization applications [20, 21].

The enormous phenotypic and genetic heterogeneity shown by the strains belonging to the *P*. *fluorescens* group [22, 23] have led into a difficult assessment of its phylogeny that does not fully encompass the taxonomy, and, the proposal that a species complex is shaping the *P*. *fluorescens* group phylogeny [23, 24]. Another difficulty within this group is the frequent description of novel species, such as *P*. *protegens* [25], and subspecies, such as *P*. *brassicacearum* subsp. *neoaurantiaca* [26], and the inclusion of strains into the *P*. *fluorescens* group, e.g. *P*. sp. UW4 [27].

Phylogenies based on the small ribosomal subunit sequence (16S rDNA gene sequence) have been one of the most common methods for phylogenetic analyses of *Bacteria*. However, this method is problematic because of its lack of resolution when comparing closely related organisms [28] as well as recombination and lateral gene transfer events [29]. Multilocus sequence analysis (MLSA) often overcomes the problems of the 16S rDNA-inferred phylogenies [30] and has proven to be more reliable than the 16S rDNA in the genus *Pseudomonas* [31], in which the sequence of three concatenated housekeeping genes (*gyrB*, *rpoD*, *rpoB*) along with the 16S rDNA has been demonstrated to work well to infer a reliable phylogeny of the *P*. *fluorescens* group [8].

With the increasing availability of genomic information, whole-genome sequence-based phylogenies or phylogenomic studies are becoming more prevalent. The advantages of phylogenomics include higher accuracy [32] than is afforded by single gene or MLSA-based phylogenies [33] as well as the possibility of using draft genomes from which 16S rDNA or housekeeping gene sequences might not be available in public databases. Several approaches have emerged in the last years to establish genome sequence-based replacements for the conventional DNA-DNA hybridization (DDH), the “gold standard” for species delineation of *Bacteria* and *Archaea* [34], either by estimating *in silico* DNA-DNA hybridization [35, 36] or by operating on a new type of scale [37, 38]. All of these methods are comprised under the term overall genome relatedness indices (OGRI) [39]. For example, the Average Nucleotide Identity (ANI) index is a popular tool to circumscribe prokaryotic species using a cut-off value of 95–96%, supported by a tetranucleotide frequency correlation coefficient (TETRA) of 0.99 [38], thought to be equivalent to the 70% DDH for species definition.

However, the Genome-to-Genome Distance Calculator (GGDC) web service [35] based on the reliable [40] GBDP (Genome BLAST Distance Phylogeny) algorithm [41] has proven to be currently the most accurate method to replace conventional DDH without mimicking its pitfalls [36], which was also the goal of ANI and is of utmost importance to ensure consistency [34, 42] in the prokaryotic species designation. The GGDC was recently improved for the delineation of prokaryotic subspecies [43]. For this study, GBDP was also used for a distance-based reconstruction of phylogenetic trees, including bootstrap support [36, 44].

Here, we compare 93 published full genomes of the *P*. *fluorescens* group strains to infer their phylogenetic relationship by using MLSA and five phylogenomic methods: a composition vector approach (CVTree) [45], a specific context-based nucleotide variation approach (Co-phylog) [46], ANIb and TETRA indices [38] and GBDP [36]. By using methods based on entire genomes, we expected to achieve a better resolution and thus to identify phylogenomic groups within this complex to compare them to the ones identified by the MLSA of type strains. ANIb and TETRA indices, along with dDDH values, were also used to establish a threshold value to obtain these groups by clustering. Finally, we have defined (i) the *P*. *fluorescens* complex core genome, (ii) the core genome of each group, (iii) the strain-specific genome, (iv) the group-specific genome, (v) the pan-genome, and (vi) the number of CDSs in each genome fraction over the number of sampled genomes by identifying clusters of orthologous CDSs. By screening the group-specific genome and searching for traits that have been described in the literature as distinctive for some species, we expected to find unique features within the groups that not only support the differentiation of the *P*. *fluorescens* group, but also allow to define the biocontrol, bioremediation and PGPR applications of these strains.

## Results and Discussion

### *“P*. *fluorescens* complex” definition

**Fig 1:**
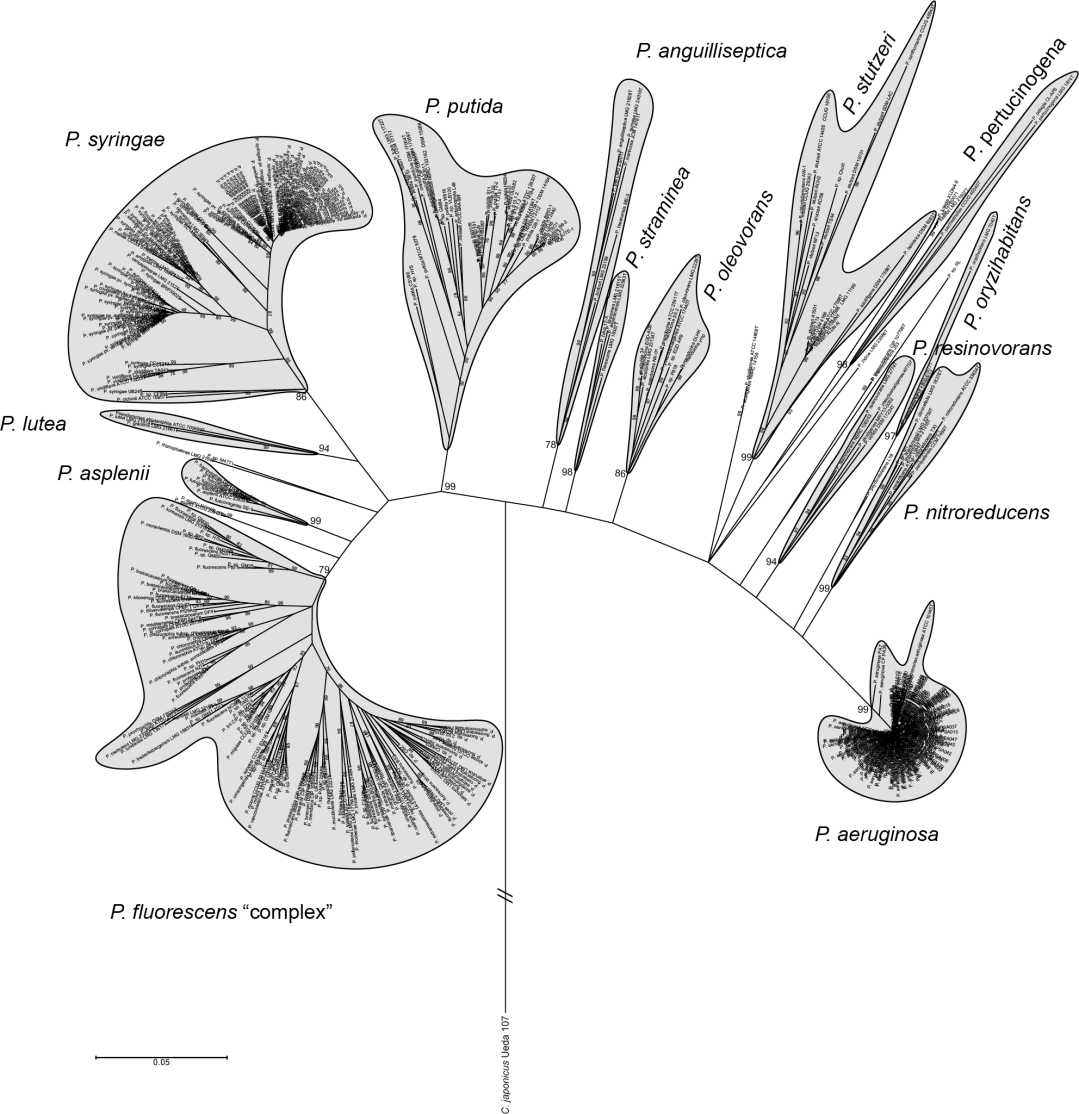
Phylogeny of the *Pseudomonas* genus inferred by MLSA. Phylogenetic tree of 451 *Pseudomonas* strains along with 107 type strains based on the concatenated partial sequences of the 16S rDNA, *gyrB*, *rpoD* and *rpoB*, ML method, Tamura-Nei. Only bootstrap values above 75% (from 1,000 replicates) are shown. *Cellvibrio japonicum* Ueda 107 was used as outgroup. Details are found in S1 Fig.

An initial MLSA tree was built with the concatenated partial sequences of 16S rDNA, *gyrB*, *rpoD* and *rpoB* genes from 451 (S1 Table) *Pseudomonas* genomes available in public databases, together with 107 type strains retrieved from the PseudoMLSA database (http://www.uib.es/microbiologiaBD/Welcome.php) and described in [8], to identify the genomes being part of the *P*. *fluorescens* group. These genes have been previously reported to be robust regarding the inference of a reliable phylogeny of this genus [8, 47]. The MLSA tree revealed the presence of 14 main groups as shown in Fig 1 (see in detail in S1 Fig), according to the topology of the tree and the presence of well-defined nodes with bootstrap support values greater than 75% over 1000 replicates. Two main lineages were identified: *P*. *aeruginosa* and *P*. *fluorescens*. The *P*. *fluorescens* lineage was composed of five groups: *P*. *putida*, *P*. *syringae*, *P*. *lutea*, *P*. *asplenii* and *P*. *fluorescens*. Several strains were not clustered in any group, including the *P*. *rhizospherae* and *P*. *agarici* type strains. Additionally, according to both the numbers of type strains and genome-sequenced strains, as well as inner-strain distances, *P*. *fluorescens*, *P*. *syringae* and *P*. *putida* were the most diverse groups within the genus (Fig 1) and the *P*. *fluorescens* group appeared to be the most distal one and exhibited an enormous diversity in both the number of species and distances within it. Although there are differences compared to previous works based on the MLSA analysis of type strains [8, 9] and both type and sequenced strains [7], where ten or eleven groups were identified, these findings were in good concordance with these previous analysis. These differences could be due to the number of strains included in our study and the clustering method.

**Fig 2:**
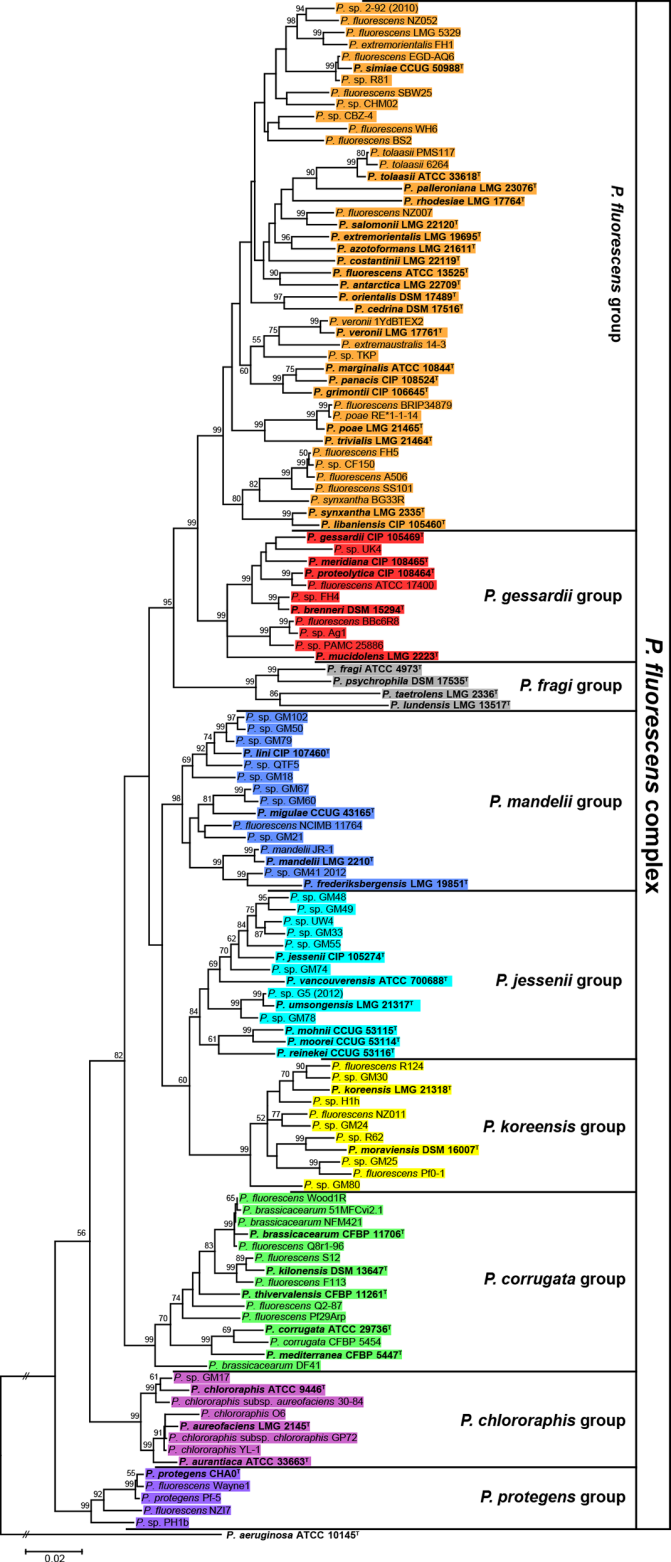
Phylogeny of the *P*. *fluorescens* complex inferred by MLSA. Phylogenetic tree of 127 sequenced and type strains belonging to the *P*. *fluorescens* complex based on the concatenated partial sequences of the 16S rDNA, *gyrB*, *rpoD* and *rpoB* genes, the ML method and the Tamura-Nei model. Only bootstrap values above 50% (from 1,000 replicates) are shown. The *P*. *aeruginosa* type strain was used as outgroup. Bold text as well as superscript ^T^ indicates type strains. Strains are colored according to the groups established in this work.

In the MLSA performed as above but with type strains and genome-sequenced strains belonging to the *P*. *fluorescens* group and using *P*. *aeruginosa* type strain as the outgroup, the group was divided into nine subgroups: *P*. *protegens*, *P*. *chlororaphis*, *P*. *corrugata*, *P*. *koreensis*, *P*. *jessenii*, *P*. *mandelii*, *P*. *fragi*, *P*. *gessardii* and *P*. *fluorescens* (Fig 2), according to the tree topology and the bootstrap support of nodes. Among these, (i) the *P*. *fluorescens* subgroup was the most distal and diverse one, while (ii) no sequenced strains were found within the *P*. *fragi* subgroup. The subgroups established here for the *P*. *fluoresccens* group were also in concordance with recent studies [7–9], although our results show that the *P*. *chlororaphis* and *P*. *protegens* subgroups are clearly separated. Given the diversity shown by the *P*. *fluorescens* group, the presence of 50 validly named species and the number of subgroups into which it was divided, henceforth we will refer to this group as the “*P*. *fluorescens* complex”, and each one of the subgroups into which it was divided will be referred to as groups.

### Phylogenomic analysis of the *P*. *fluorescens* complex

**Fig 3:**
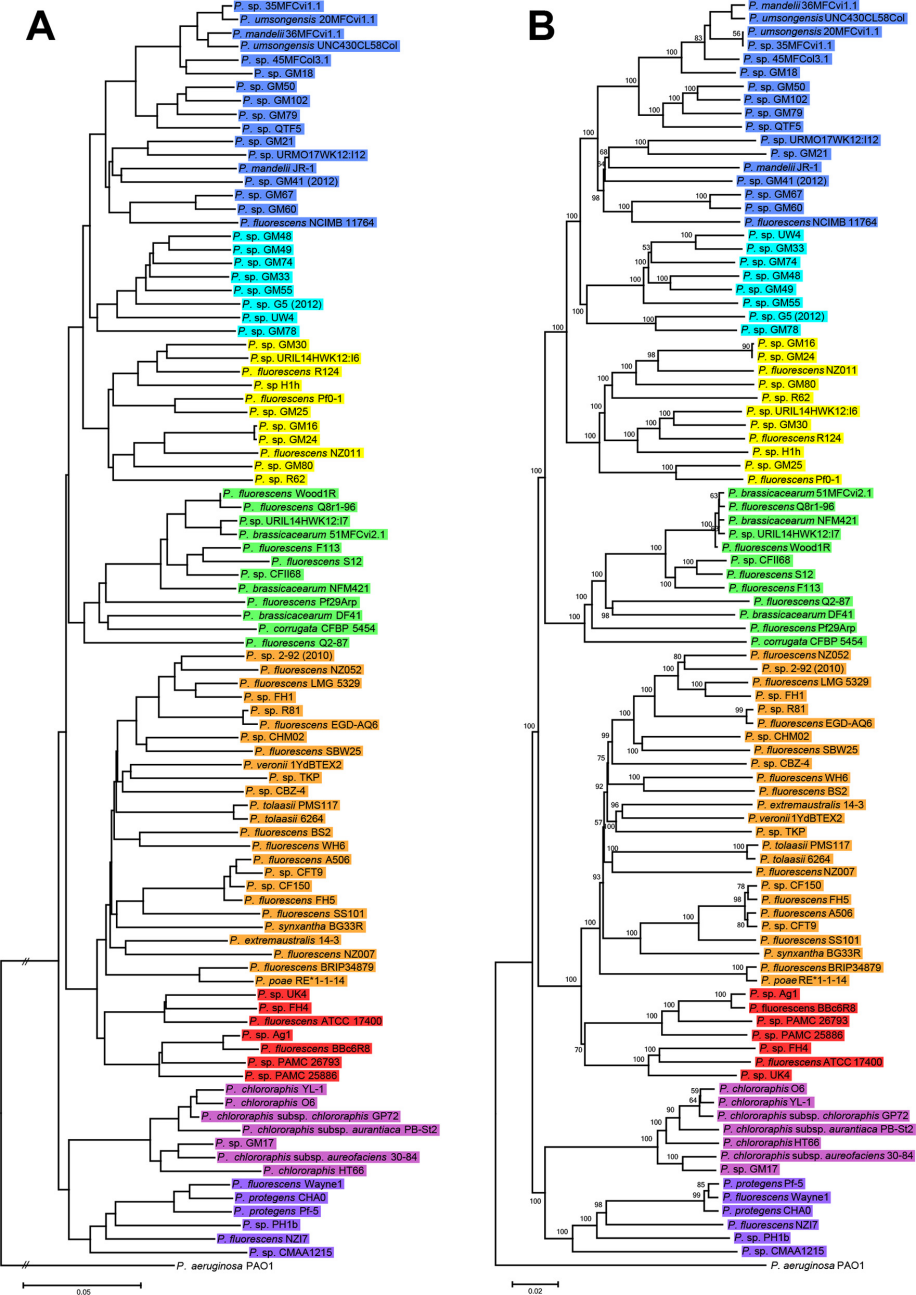
Phylogeny of the *P*. *fluorescens* complex based on ANIb and GBDP. Whole-genome phylogenies based on nucleotide data from 93 sequenced strains belonging to the *P*. *fluorescens* complex. Strains are colored according to the groups established in this work. The left-hand phylogeny (A) was inferred by using ANIb values converted to distances (100 –ANIb % similarity) calculated with BLAST algorithm and the NJ method. The right-hand phylogeny (B) is based on all pairwise intergenomic distances as calculated by the latest GBDP version and inferred using FastME v2.07 with TBR postprocessing. Only greedy-with-trimming pseudo-bootstrap values above 50% (from 100 replicates) are shown. For both phylogenies *P*. *aeruginosa* PAO1 was used as outgroup.

Although MLSA approach is practical for establishing groups within strains, housekeeping genes belong to the core genome. Phylogenomic approaches provides additional information on using genes that could potentially lead to significant differences between strains (phenotypical and ecological characteristics), and could probably justify their separation into distinct species or ecotypes [30, 32]. Several methods exist that can estimate distances from completely or partially sequenced genomes (e.g. CVTree [45], Co-phylog [46], ANIb index [38] and GBDP [36]). These methods were used to infer the phylogeny of the *P*. *fluorescens* complex genomes. The ANIb and nucleotide GBDP output trees were topologically similar (Fig 3), showing eight well-defined phylogenomic groups that correlated with those identified by MLSA. These groups were also observed in the output trees built with Co-phylog, a context-based nucleotide variation approach (S2 Fig), and the use of aminoacid sequences in CVTree and GBDP (S3 Fig). However, there were differences in the relative position of the groups compared to our MLSA (Fig 2) and also minor differences between all the trees in the strains order within these groups. Among the different methods used, only GBDP (Fig 3B) yields branch support values and thus allows for distinguishing between groups really supported by the data and those occurring by chance alone. All in all, this led to the identification of eight groups that are in concordance with the MLSA analysis. For the *P*. *fragi* group, as shown in the MLSA tree (Fig 2), there were no genome sequence data available at all and, therefore, was not present in any of the phylogenomic trees.

The concordance between the grouping obtained with MLSA (a selection of core-genome genes) and the phylogenomic methods (full genomes) indicate co-evolution of the core and the accessory genome, and therefore that similar selective pressure is shaping both parts of the genome and it is likely that phylogenomic groups also represent eco-physiological groups. These results also support the proposals of using these housekeeping genes for routine identification of *Pseudomonas* isolates [48, 49]. It has also been shown that MLSA correlates with ANIb and GBDP in the genus *Pseudomonas* [7]. Previous phylogenomic studies with ten and eleven sequenced strains belonging to this complex resulted in the identification of three [22] and four groups [50] respectively, whereas a more recent phylogenomic evaluation of 50 sequenced strains has led into the identification of five groups [10]. These results compared with our phylogenomic analysis of 93 sequenced strains, provide evidence that the phylogenetic organization of the *P*. *fluorescens* complex is not currently fully defined and it is likely to change as new strains and genomic information is available, as previously suggested [24]. Although all the phylogenomic trees inferred with nucleotide sequences (Fig 3 and S2 Fig) are similar, some differences in topology appear when compared with the ones inferred from aminoacid sequences (S3 Fig). These differences were well supported with GBDP, as shown by bootstrap values (S3 Fig), and were also noticed in previous works [10, 50]. This discrepancy is probably due to codon degeneracy and the resulting aminoacidic sequence conservation. Nevertheless comparison of aminoacidic against nucleotide variation provides information of the degree of selective pressure on each strain.

### Genome clustering of digital DDH, ANIb and TETRA

**Fig 4:**
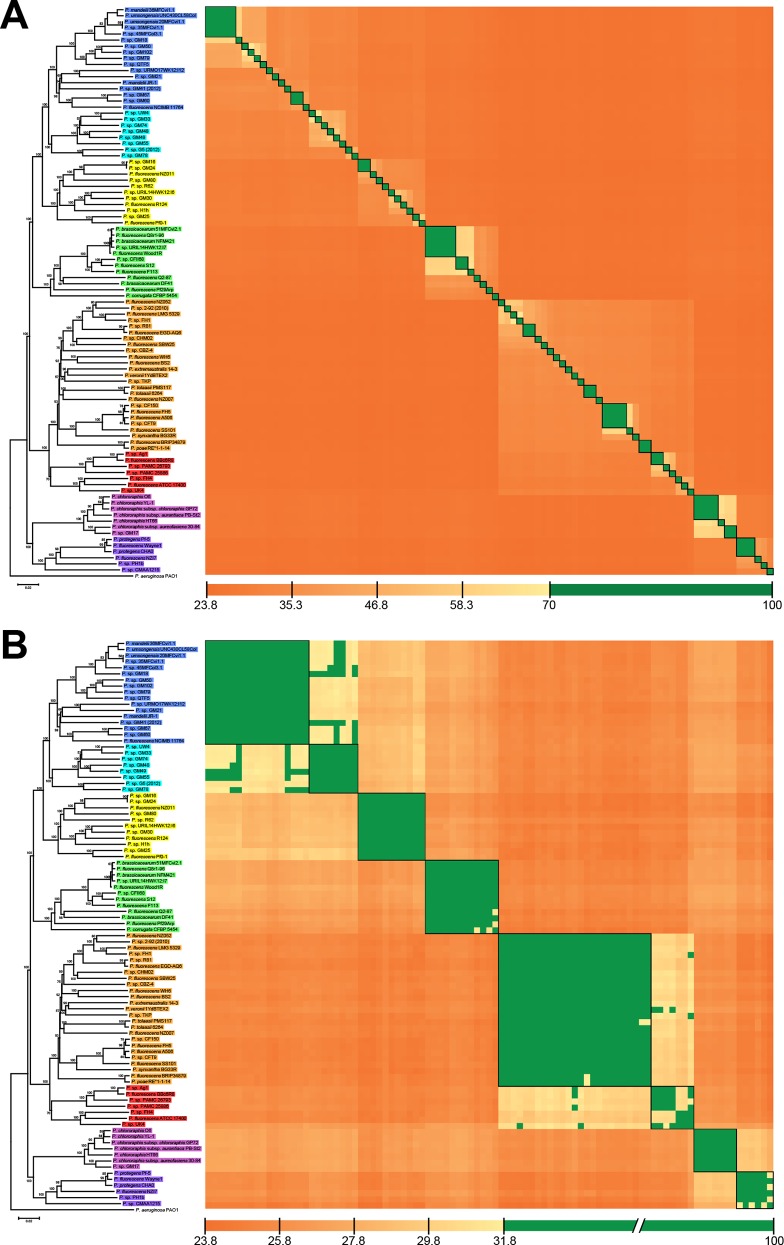
Heatmap of reciprocal dDDH values under species and groups thresholds. (A) Species-level clusters at a dDDH threshold of 70% and (B) group-level clusters at 31.8% dDDH threshold. The phylogenomic tree from Fig 3B was used for ordering strains in the symmetric matrix. Black boxes on the heatmap matrix represent species or group clusters according to the OPTSIL results.

Currently two methods have been proposed to be an *in silico* replacement for the DDH “gold standard” for prokaryotic species boundaries. These comprise the GGDC algorithm for calculating dDDH [35] and various implementations of the average nucleotide identity [37, 51, 52] including ANIb with a cut-off value of 95–96% (and additional support by TETRA > 0.99) [38]. Our results with dDDH calculations between all the sequenced strains within the *P*. *fluorescens* complex and using the OPTSIL clustering algorithm revealed that in the 93 tested strains, 69 species clusters (according to 70% dDDH) and 75 subspecies clusters were identified, whereas ANIb yielded a variable number from 64 to 71 (depending on the applied threshold of 95–96%) species-level clusters can be discriminated (S2 Table). Aside from OPTSIL clusters, dDDH values (S3 Table) were also examined in a heatmap matrix, where the 69 species-level clusters were clearly visualized (Fig 4A). The 69 clusters determined with dDDH data were also found using an ANIb threshold of 95.7% (S2 Table). However, given that, of the 50 type strains included in the *P*. *fluorescens* complex, only the type strain *P*. *protegens* CHA0 [53] was sequenced and available in our dataset, it was taxonomically impossible to determine whether these species-level clusters belonged to a previously described species or if they should be considered as new species. The sequenced type strain *P*. *protegens* CHA0 allowed us to determine that this strain, along with *P*. *protegens* Pf-5 and *P*. *fluorescens* Wayne1, are all clearly belonging to the same species according to the dDDH criterion (90.9% on average). In contrast, ANIb yielded inconsistent results regarding this group because the single comparison of *P*. *protegens* Pf-5 and *P*. *protegens* CHA0 resulted in an ANIb value below the 95% species boundary (S3 Table). On the contrary, according to the species clusters as calculated via OPTSIL (S2 Table), these three strains were assigned to a single species cluster under both the dDDH and ANIb parameter, which is explained by the applied fraction of links required for cluster fusion *F* (0.5, i.e., average-linkage clustering), which was previously shown to yield optimal clustering results [43]. These inconsistencies in ANIb values were also found in strains of the *P*. *chlororaphis*, *P*. *corrugata* and *P*. *mandelii* groups while dDDH values did not show any discrepancy for establishing species-level clusters, as shown in Fig 4A. This results were not unexpected as only GBDP has been shown to almost always result in consistent distance matrices at the species threshold [54] In any case, one can also not exclude an effect on the clustering, when more type strain genomes are added.

To cluster the genomes into the eight groups within the *P*. *fluorescens* complex identified by our MLSA and phylogenomic analysis, OPTSIL was used to find the best clustering parameters: distance threshold *T* and fraction of links required for cluster fusion *F* (S4 Table). Regarding dDDH, full agreement according to Modified Rand Index (MRI = 1) with the reference partition was found for F = 0.25 and T = 0.132915, which equals 31.8% dDDH. This dDDH threshold was also represented in a heatmap matrix (Fig 4B), showing the eight phylogenomic groups, although some strains were not in full agreement with the OPTSIL clustering, which is explained by the applied *F*. ANIb was found to have a less than optimal agreement, with the highest MRI (0.97) found under the clustering parameters *F* = 1 and *T* = 0.152325, representing 84.8% ANIb. Here, the disagreement to the reference partition was caused by the *P*. *koreensis* group, which was effectively divided into two clusters (S2 Table). Therefore, taking into account dDDH, ANIb and TETRA the *P*. *fluorescens* complex can only be reliably clustered into the eight phylogenomic groups using the dDDH approach.

**Fig 5:**
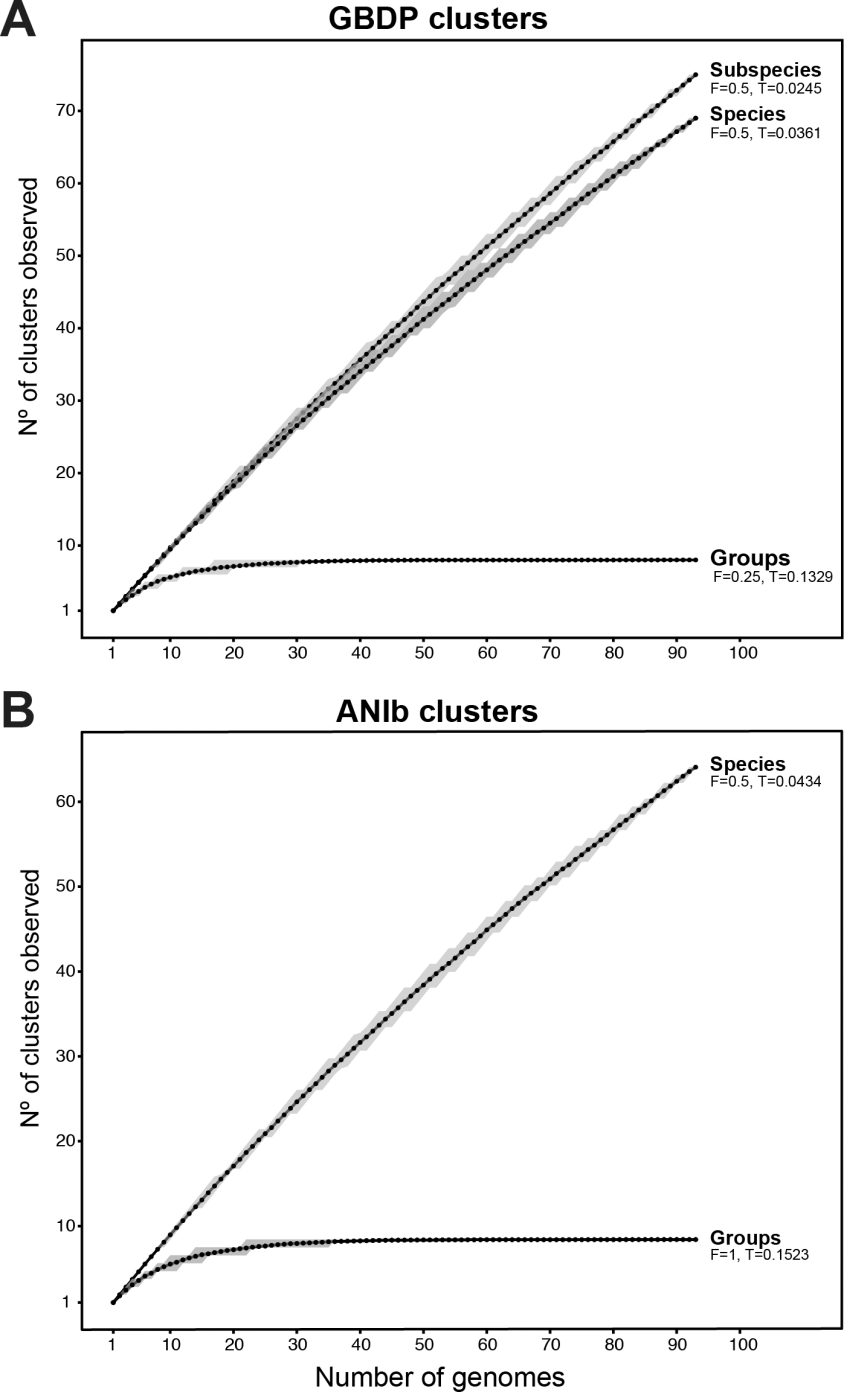
Simulation of the variation in several clusters dependent on the number of genomes. (A) Number of GBDP dDDH clusters observed for subspecies, species and groups over the number of randomly sampled *P*. *fluorescens* complex genomes. (B) Number of ANIb clusters observed for species and groups over the number of randomly sampled *P*. *fluorescens* complex genomes. Clusters were established under the best OPTSIL parameters. Black dots indicate the mean of the calculated values, while gray shades indicate the first and third quartile.

Cluster and triplet consistency over the complete ranges of *T* and *F* were also assessed for dDDH and ANIb (S1 File). At the relevant thresholds for the limitation of species and the eight phylogenomic groups for GBDP and ANIb, GBDP yielded the highest mean consistency values for both, species and phylogenomic groups (S1 File). We further represent these OPTSIL clusters under the best thresholds in dDDH and ANIb in collector’s curves as a function of the number of clusters observed over the number of genomes randomly sampled from 200 replicates. Regarding dDDH (Fig 5A), subspecies and species curves are far from reaching an asymptote and it is obvious that as new strains are isolated, the number of species-level clusters is going to increase to reach a value in the hundreds, as it does not seem near to reaching an asymptote. Conversely, using the OPTSIL threshold for group clusters (corresponding to a 31.8% dDDH) show eight groups, which is in agreement with our MLSA and phylogenomic analyses and demonstrates that any combination of the 30 given genomes provide enough information to define all the phylogenomic groups within the *P*. *fluorescens* complex. Although a similar curve was observed with ANIb data (Fig 5B), here the best OPTSIL threshold for group identification showed the presence of 9 groups caused by the division of the *P*. *koreensi* group into two clusters. Therefore, the number of sampled genomes required to achieve this number of clusters is around 40 genomes (Fig 5B). These results also show that it is unlikely that the number of phylogenetic groups established at this level will grow much further in the future. This number of groups is in good accordance with the results of Gomila *et al*. [7], Mulet *et al*. [8, 9], and others [10, 22] with a lower number of sequenced genomes. The results presented here show that beside their use for determining species boundaries, only GBDP can be used to safely determine higher-level phylogenetic relations and to discriminate between these groups within the *P*. *fluorescens* complex.

### Core, group-specific, strain-specific and pan-genome analysis

The core genome consists of the orthologous coding sequences (CDSs) found in all the strains belonging to a cluster, either to the entire *P*. *fluorescens* complex or to each of the eight groups within this complex. The group-specific genome is defined as the set of orthologous CDSs found in the core genome of each group that are not found in the core genome of any other group. The strain-specific genome consists of the singletons and paralogous CDSs found in a strain and not shared with any other strain. Finally, the pan-genome consists of the full set of orthologous CDSs, paralogous CDSs and singletons found in all the strains within the *P*. *fluorescens* complex, including the strain-specific genome.

**Fig 6:**
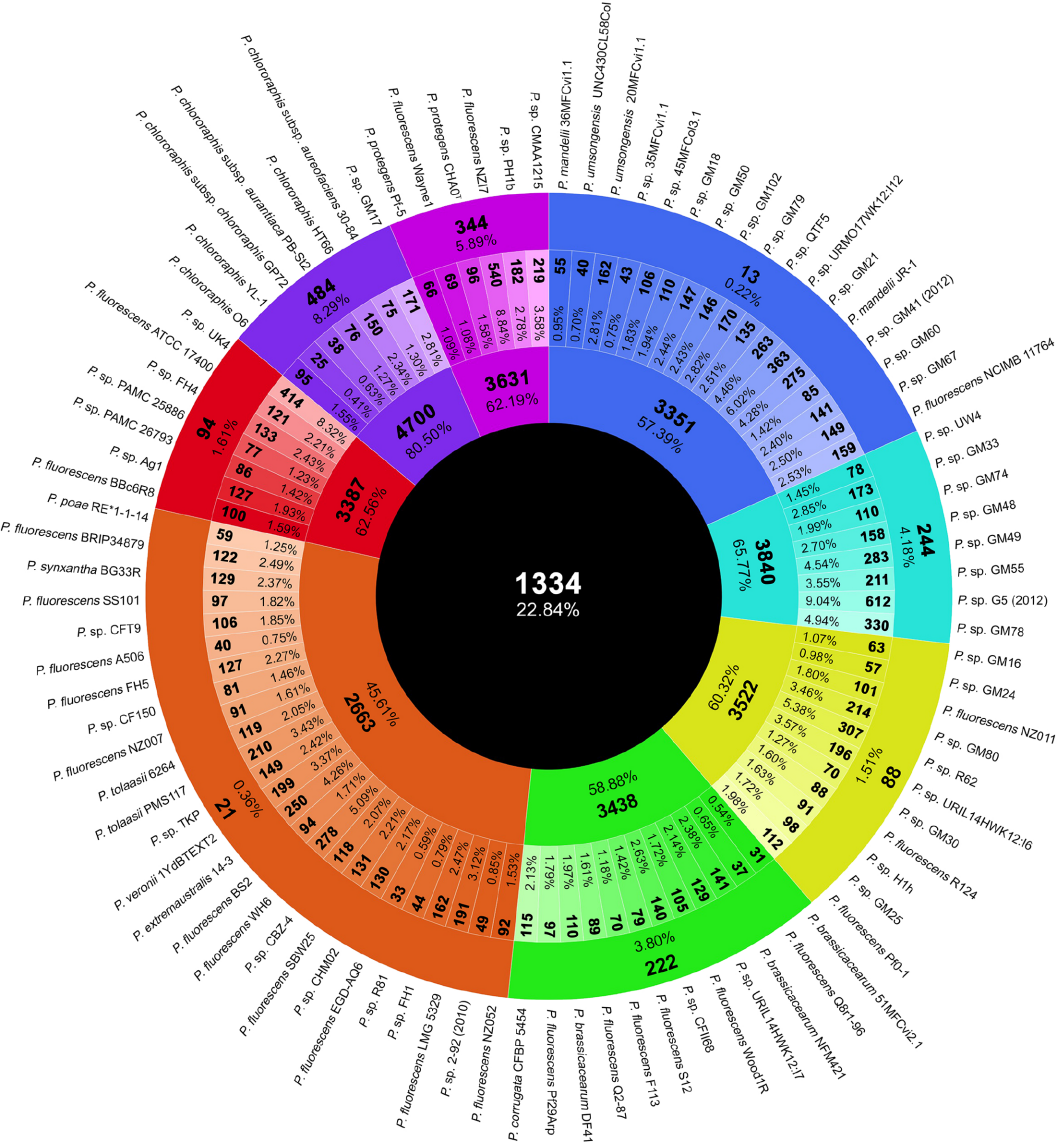
Number and percentages of orthologous CDSs belonging to the core, strain-specific and group-specific genome. From outside to inside, the outer most circle shows the strain names of the *P*. *fluorescens* complex used in this work. The second circle represents the number of group-specific orthologous CDSs (bold) and the percentage it represents from the average of CDSs in all the genomes. The third circle indicates the strain-specific number of CDSs (bold) and its percentage relative to the total number of CDSs from each strain. The fourth circle represents the number of CDSs (bold) in the core-genome of each group and its percentage of the average number of CDSs in all the genomes. The fifth circle represents the core-genome CDSs of the *P*. *fluorescens* complex and their percentages of the average number of CDSs in all the genomes. Coloring is according to the groups established in this work.

The evaluation of the orthologous CDSs shared between all the 93 strains within the *P*. *fluorescens* complex revealed a small core genome composed of 1,334 CDSs, representing 22.84% of the genomes average CDSs (5839 CDSs) (Fig 6). Previous analysis with three (3,642 CDSs), nine (2,781 CDSs), ten (2,789 CDSs) and 50 (2,003 CDSs) *P*. *fluorescens* complex strains [10, 22–24] showed larger core genomes, which is expected as they contain a lower number of strains. The core genome of this complex is considerably smaller than in *P*. *aeruginosa*, and similar to the ones in *P*. *syringae* and *P*. *putida* represented by five strains each [22]. Compared with other genera, the *P*. *fluorescens* complex core genome is smaller than the one calculated for 186 *Escherichia coli* genomes, which consists of 1,702 homologous gene clusters [55]. It is also remarkably smaller than the 3,884 CDSs found in the core genome of eight *Pantoea ananatis* strains, which seemed to have reached the asymptote [56]. The small core genome of the *P*. *fluorescens* complex is probably a consequence of its high diversity and genomic heterogeneity. On the other hand, the group core genome ranges from 2,663 to 4,700 CDSs (groups *P*. *fluorescens* and *P*. *chlororaphis* respectively), representing from 45.61 to 80.50% of the genomes average CDSs. As expected, the core genomes of each of the eight groups are bigger than the complex core genome (Fig 6). Only a single previous study calculated the core genome of the *P*. *corrugata* group, as established here, with five strains resulting in 4,407 CDSs [10]. In our analysis, the core genome of this group was smaller (3,438 CDSs), likely due to the larger number of strains included. Regarding the strain-specific genome (140 CDSs on average), this genomic fraction is more variable, ranging from 25 to 612 CDSs (*P*. *chlororaphis* YL-1 and *P*. sp. G5 (2012) respectively) and representing from 0.41 to 10.48% of the genomes average CDSs (Fig 6). Similar numbers can be found in another work regarding strains within the *P*. *fluorescens* complex [10]. Finally, the group-specific genome calculated for each of the eight groups varies from 13 CDSs in *P*. *mandelii* to 484 CDSs in *P*. *chlororaphis* groups, representing from 0.22 to 8.29% respectively of the genomes average CDSs (Fig 6). *P*. *mandelii* and *P*. *fluorescens* groups have the smallest group-specific genome (13 and 21 CDSs respectively), which is in concordance of the larger number of strains the groups include, and suggest that the genome of these groups could resemble the ancestral organism that led to the diversity of the rest of the groups.

**Fig 7:**
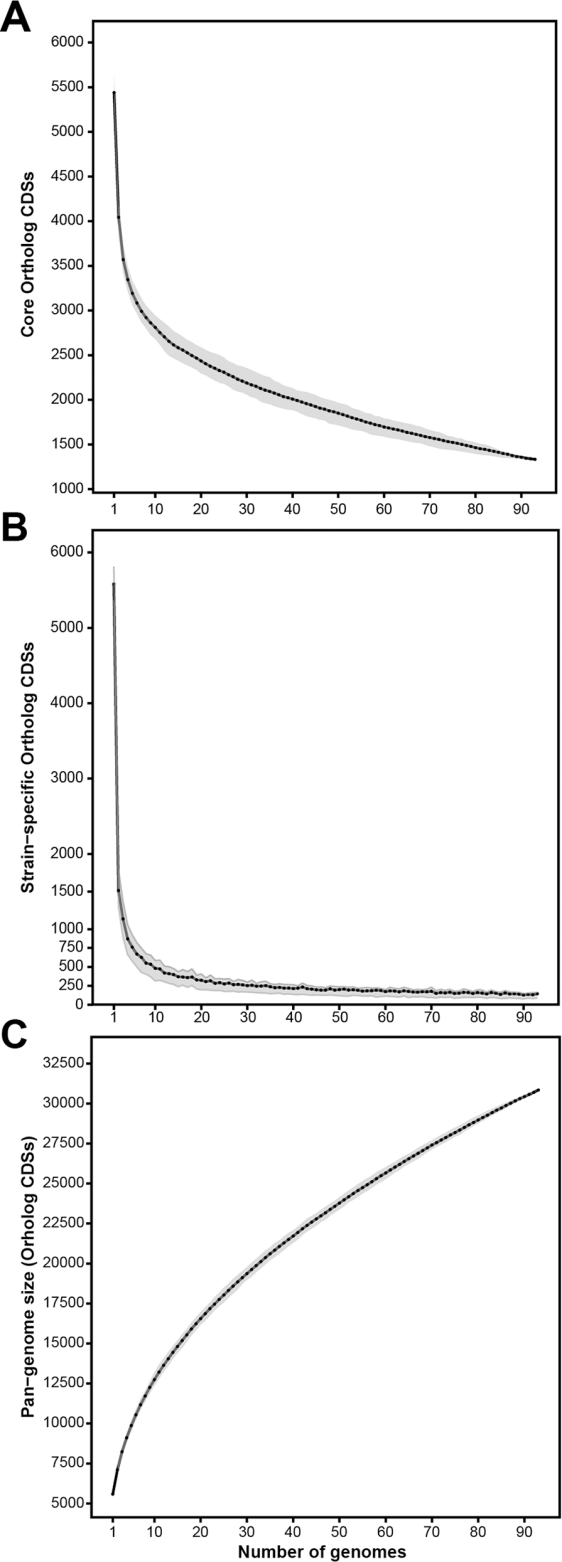
Simulations for the variation in core, strain-specific and pan-genome fractions over the number of genomes. (A) Number of core CDSs depending on the number of genomes sampled. (B) Number of strain-specific CDSs depending on the number of genomes sampled. (C) Pan-genome size (CDSs) depending on the number of genomes sampled. In all cases, genomes were sampled with 200 replicates. Black dots indicate the mean of the calculated values, while grey shades indicate the first and third quartile.

To test genome variability, simulations were carried out based on the fluctuations of shared CDSs either in the core genome, strain-specific genome or pan-genome over sequentially added strains (Fig 7). As shown in Fig 7A, the *P*. *fluorescens* core genome dramatically decreases with the first ten randomly added strains, and then gradually reduces further with the addition of new strains. With more than 90 strains, the rate of decrease is clearly slowed down and seems almost asymptotic. This curve is in agreement with the core-genome calculations in previous works with lower number of strains from the *P*. *fluorescens* complex strains [10, 22–24]. Similar behavior is seen in the curve showing the strain-specific CDSs as a function of the number of new CDSs observed over sequentially added strains (Fig 7B). The first ten randomly chosen strains are enough to cover most of the genetic diversity and, therefore, more genomes will only add their strain-specific genome (140 CDSs average), which is congruent with the identification of eight groups within *P*. *fluorescens*. Although the core-genome and strain-specific genome curves are similar, it is important to notice that while strains keep adding specific CDSs, core-genome size might be modified.

The genetic diversity shown by these models is further reflected in the pan-genome, comprising 30,848 CDSs, representing on average 5.28 genomes (Fig 7C). This pan-genome analysis is in agreement with previous calculations [22, 24], and it is remarkably larger than the ones estimated for five *P*. *aeruginosa* strains (7,824) [22] and 19 *P*. *syringae* strains (12,829) [57]. Compared with other genera, the pan-genome of the *P*. *fluorescens* complex is more than twice the size of the *E*. *coli* [55] and the *P*. *ananatis* [56] pan-genomes, and slightly larger than the one calculated for the *Epsilonproteobacteria* inter-species genomes [58]. These differences can be explained by the diversity shown by the *P*. *fluorescens* complex. Our plot (Fig 7C) also shows that it is an “open” pan-genome, as with 93 strains it does not reach an asymptote and newly added strains will substantially increase the pan-genome size.

### Phylogenetic distribution of specific traits

**Fig 8:**
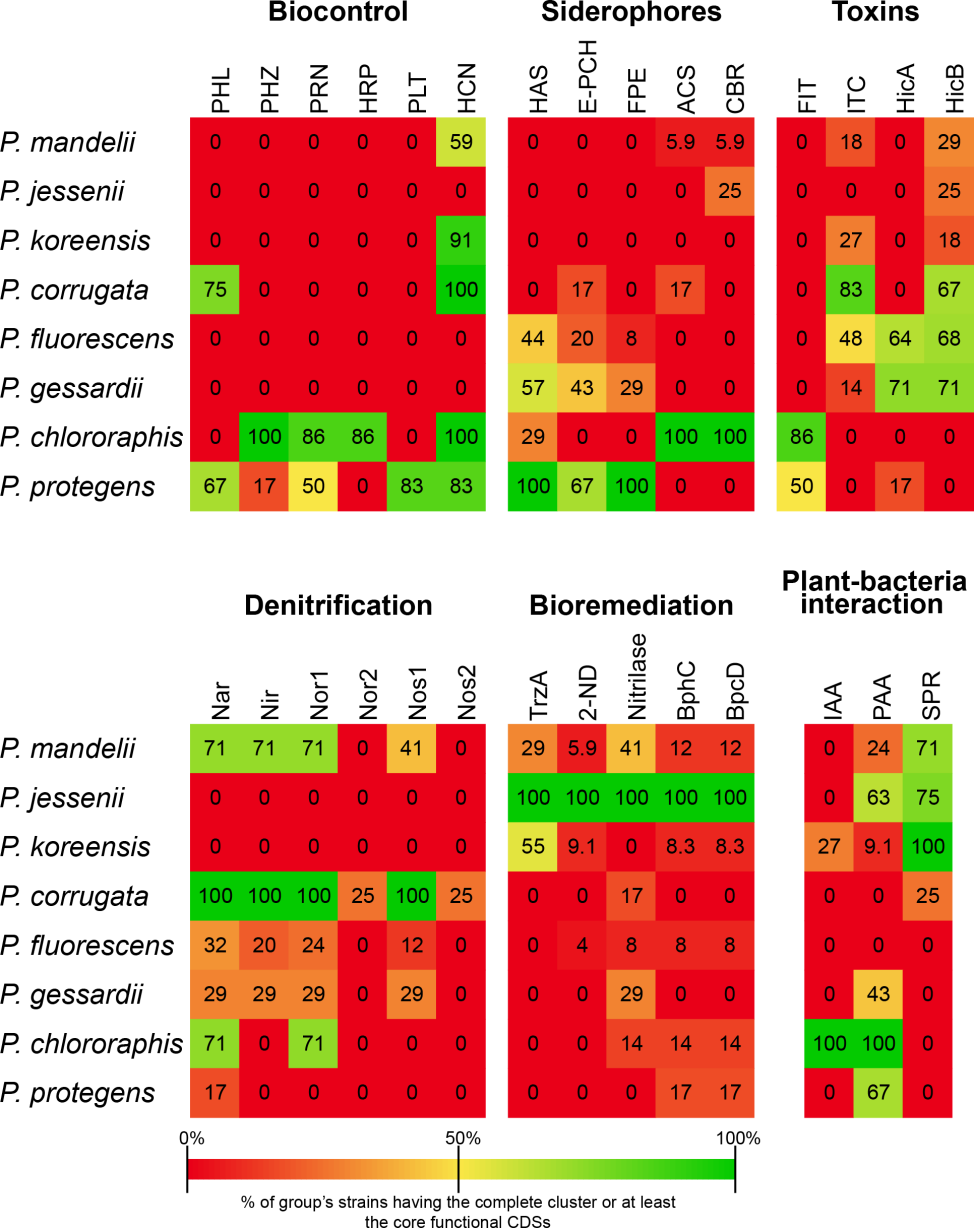
Distribution of the selected group-defining clusters in the groups from the *P*. *fluorescens* complex. Numbers within the boxes represent the percentage of strains from each group having the complete gene cluster or at least the core functional CDSs. PHL: DAPG (2,4-diacetylphloroglucinol) biosynthesis (PhlABCDEFGH); PHZ: phenazine biosynthesis (PhzABCDEFGOIR); PRN: Pyrrolnitrin biosynthesis (PrnABCD); HRP: 2-hexyl, 5-propyl resorcinol biosynthesis (DarABCRS); PLT: Pyoluteorin biosynthesis (PltABCDEFGIJKLMNOPRZ); HCN: hydrogen cyanide (HcnABC); HAS: Hemophore biosynthesis (HasADEFIRS); E-PCH: Enantio-pyochelin biosynthesis (PchABCDEFHIKR); PFE: Ferric- enterobactin receptor (PfeARS); ACS: Achromobactin biosynthesis (AcsABCDEF+YhcA); CBR: Achromobactin transport (CbrABCD); Nar: Nitrate reductase (NarGHIJKLUX); Nir: Nitrite reductase (NirCDEFGHJLM1NOPS+NorQ); Nor: Nitric oxide reductase (NorBCD); Nos: Nitrous oxide reductase (NosDFLRYZ); FIT: FitD toxin (FitABCDEFGH); ITC: Insecticidal toxin complex; 2-ND: 2-nitropropane dioxigenase; IAA: Indole-3-acetic acid metabolism (IaaMH); PAA: Phenylacetic acid catabolism (PaaABCDEFGHIJKLNWXY); SPR: spermidin biosynthesis (S-adenosylmethionine decarboxylase and spermidine synthase).

The specific genome of the eight established groups was screened to identify group-specific features. Several traits, previously described in the literature and representative of species or species groups belonging to the *P*. *fluorescens* complex [22, 24] were also examined. Although most of the group-specific CDSs were annotated as hypothetical proteins, we could describe several important clusters of orthologous CDSs phylogenetically distributed and even specific to groups within the *P*. *fluorescens* complex. We have divided these clusters into six categories: biocontrol, siderophores, denitrification, toxins, bioremediation and plant-bacteria interaction. These clusters are summarized in Fig 8 and are discussed below. The presence of each of the proteins within the clusters in each strain can further be seen in S2 File.

#### Biocontrol

Fluorescent *Pseudomonas* strains are known to contribute to soil disease suppression and to protect plants from soil-borne fungal and bacterial pathogens by the production of a huge array of secondary metabolites, including antibiotics and fungicides [3, 14–16]. The cluster for the biosynthesis of the antifungal polyketide 2,4-diacetylphloroglucinol (DAPG) is present in nine strains within the *P*. *corrugata* group and in four strains belonging to the *P*. *protegens* group. This genetic cluster is absent from all the other groups. Biological evidence of DAPG production has been previously reported in *P*. *fluorescens* F113 [59], *P*. *fluorescens* Q2-87, *P*. *fluorescens* Q8r1-96 [60], *P*. *protegens* Pf-5 and *P*. *protegens* CHA0 [25]. DAPG producers have been shown to be highly effective biocontrol agents against a variety of plant diseases [16]. Pyoluteorin is a known toxin against oomycetes [61], certain bacteria and fungi [62] and, at high concentrations, exhibits phytotoxicity against certain plants [63]. It is produced by several *Pseudomonas* species, including *P*. *aeruginosa* [64]. Within the *P*. *fluorescens* complex, five *P*. *protegens* group strains harbor the biosynthetic cluster for pyoluteorin biosynthesis, previously described in *P*. *protegens* Pf-5 [65, 66]. These genes are not present in any other strain from other groups within the *P*. *fluorescens* complex and thus seem to be a trait specific to the *P*. *protegens* group. On the other hand, all the *P*. *chlororaphis* strains and one *P*. *protegens* strain (*P*. sp. PH1b) harbor the cluster for the biosynthesis of phenazine-1-carboxylic acid (PCA) and five strains also have the *P*. *chlororaphis* exclusive PhzO protein, which produces 2-hydroxyphenazine-1-carboxylic acid (2OHPCA), from which 2-hydroxyphenazine (2OHPZ) is spontaneously formed [67]. The production of phenazines has important roles in biocontrol of a wide range of plant pathogenic fungi. Phenazines produced by *P*. *chlororaphis* have shown to inhibit soil-borne phytopathogenic fungi [68, 69] and contribute to the natural suppression of *Fusarium* wilt disease [70]. Furthermore, phenazines have been shown to activate the induced systemic resistance in plants [71]. The results presented here show that phenazines production is a common trait of the *P*. *chlororaphis* group, although occasional appearances in other groups might occur. Six strains from the *P*. *chlororaphis* group (but no other strain) also contain the biosynthetic cluster for the antifungal 2-hexyl, 5-propyl resorcinol, which has previously been identified in two *P*. *chlororaphis* strains [72, 73]. It has been reported, that this compound has moderate antifungal and antibacterial properties [74, 75]. *P*. *chlororaphis* along with the closely related *P*. *protegens* strains share the cluster for the biosynthesis of the biocontrol siderophore pyrrolnitrin [76]. This finding is in agreement with previous reports of pyrrolnitrin production in strains from these groups [22]. Pyrrolnitrin is known to produce an inhibitory effect on the electron transport system in fungi, and it displays a wide range of antifungal activity [20]. Finally, the cluster for the hydrogen cyanide production, a known volatile toxic against nematodes [77], was found in all the strains from *P*. *corrugata* and *P*. *chlororaphis*, and in a certain number of strains from *P*. *protegens*, *P*. *koreensis* and *P*. *mandelii* groups. All together, these findings show that strains belonging to *P*. *corrugata*, *P*. *chlororaphis* and *P*. *protegens* groups would be particularly suited for biocontrol applications.

#### Siderophores

Soil bacteria are known to produce diverse siderophores to sequester iron from the environment through high-affinity interactions [78]. Iron exists primarily in the insoluble ferric oxide form [79], which is not available to microorganisms. Unsurprisingly, we identified different clusters of orthologous CDSs involved in the biosynthesis of several iron-siderophores. A hemophore-dependent heme acquisition biosynthetic and transport cluster is present in all the strains from the *P*. *protegens* group, and is also distributed among certain strains from the *P*. *chlororaphis*, *P*. *fluorescens* and *P*. *gessardii* groups. This heme acquisition system is able to capture heme from a wide range of substrates, and it has been suggested that it could also secure heme from other organisms [80, 81]. This suggests that strains from these groups could have a competitive advantage in iron-limited environments. Another iron-chelating siderophore we have identified in certain strains from the *P*. *protegens*, *P*. *gessardii*, *P*. *fluorescens* and *P*. *corrugata* groups is enantio-pyochelin. This cluster has previously been reported in *P*. *protegens* CHA0, where it has been shown that the (enantio-)pyochelin-mediated iron uptake machinery is stereospecific [82]. This is believed to be a means of preventing potential competitors occupying the same ecological niche from stealing heterologous iron-siderophore complexes [83]. A ferric-enterobactin receptor has also been found in all the strains from the *P*. *protegens* group, and some strains from the *P*. *gessardii* and *P*. *fluorescens* groups. It has been proposed for a *P*. *putida* strain carrying this system but not the enterobactin biosynthetic cluster [84], that it might be more competitive against *Enterobacteria* for iron by utilizing the ferric enterobactin complex [2]. All the strains within the *P*. *chlororaphis* group also harbor the biosynthetic and transport clusters for the iron-uptake siderophore achromobactin, and, although both clusters are also present in certain strains from *P*. *mandelii* group, some *P*. *corrugata* genomes encode only the biosynthetic cluster. The description of endophytic *P*. *chlororaphis* strains [85, 86] makes achromobactin an important feature for colonizing plant internal tissues, as iron seems to be severely restricted in the plant extracellular environment [87]. On the other hand, known plant pathogens, such as *P*. *syringae* strains and *Erwinia chrysanthemi* produce achromobactin during plant infections [88, 89]. It has been proven that achromobactin is not required for causing the pathogenicity [89], suggesting achromobactin plays an important role in iron-limiting conditions, in either pathogenic or PGPR bacteria.

All of these results show that most of the strains belonging to the *P*. *protegens*, *P*. *chlororaphis*, *P*. *gessardii* and *P*. *fluorescens* groups harbor a set of iron siderophores that seems to be essential under iron-limiting conditions. The presence of several siderophores that are able to secure iron from other organisms also highlights the rhizo-competence properties of these groups.

#### Denitrification

Denitrification ability has previously been reported in *P*. *fluorescens* F113 [10], and some of the genes involved in this feature have also been found in other strains of the *P*. *fluorescens* complex [10], although it is not exclusive to strains of the *P*. *fluorescens* complex [90, 91]. The denitrification pathway is composed of four enzymatic activities: nitrate reduction (NO_3_^-^ → NO_2_^-^) by the *nar* gene cluster, nitrite reduction (NO_2_^-^ → NO) by the *nir* gene cluster, nitric oxide reduction (NO → N_2_O) by the *nor* gene cluster and nitrous oxide reduction (N_2_O → N_2_) by the *nos* gene cluster [92]. The products of these four clusters are present in all the strains from the *P*. *corrugata* group and in seven, two and one strains from the *P*. *mandelii*, *P*. *gessardii* and *P*. *fluorescens* groups, respectively. We also found that three strains from the *P*. *corrugata* group also harbor a second Nor and Nos clusters (*P*. *fluorescens* F113, *P*. *brassicacearum* DF41 and *P*. *brassicacearum* NFM421). The presence of two Nor and Nos clusters might indicate higher denitrification levels.

All these data indicate that denitrification is a defining feature of the *P*. *corrugata* group and could be related to competitive colonization of the rhizosphere, as shown previously [93]. It is also important to notice that Nar, Nir and Nor clusters are present in strains of the other groups with the exception of *P*. *jessenii*, *P*. *koreensis* and *P*. *protegens*. It is likely that denitrification is related to colonization of the rhizosphere, as mutants affected in nitrate and nitrite reductases are generally deficient in rhizosphere colonization and competitiveness [94–96].

#### Toxins

Florescent pseudomonads have been shown to have insecticidal activities towards agricultural pest insects [97] and other insects [98, 99]. The Fit insect toxin cluster is only present in strains from the *P*. *chlororaphis* and *P*. *protegens* groups. This cluster was first identified in *P*. *protegens* Pf-5, in which the production of this toxin has been associated with the lethality of this strain against the tobacco hornworm *Manduca sexta* [100]. The complete gene cluster has also been identified in *P*. *protegens* CHA0 [101] and *P*. *chlororaphis* strains O6 and 30–84 [22], suggesting Fit toxin is probably a common characteristic exclusively shared by these two closely related groups. We have also identified a cluster of insecticidal toxins, mainly present in *P*. *corrugata* group strains, but also in a few strains from *P*. *fluorescens*, *P*. *koreensis*, *P*. *mandelii* and *P*. *gessardii* groups. In fact, except the *P*. *jessenii* group, in which we could not identify any potential insecticidal toxin, strains from all the groups harbor them. These toxins are more prevalent in strains from the *P*. *corrugata*, *P*. *chlororaphis* and *P*. *protegens* groups, enhancing the potential application of strains from these groups in biocontrol of agricultural pest insects. We also identified the HicAB toxin-antitoxin system in most of the strains from the *P*. *gessardii* and *P*. *fluorescens* groups. In *E*. *coli*, it has been shown that HicA is an inhibitor of translation, and HicB is a protein that neutralizes HicA [102]. Although it has been reported that the HicAB system is widely distributed among *Bacteria* and *Archaea*, including *P*. *syringae* [103], the exclusivity of this system in certain groups of the *P*. *fluorescens* complex might suggest the basis for a competitive advantage in certain conditions. More interestingly, a different number of strains from all the groups except *P*. *chlororaphis* and *P*. *protegens* only harbor HicB, suggesting these strains could neutralize HicA toxicity expressed by other strains.

#### Bioremediation

Certain *P*. *fluorescens* strains are able to detoxify organic and inorganic pollutants, combating heavy metal pollution and pesticide bioremediation [104]. We have identified an s-triazine hydrolase that catalyzes both the dechlorination and deamination reactions of diamino-s-triazines such as desethylsimazine and desethylatrazine [105] in all the strains of the *P*. *jessenii* group. The triazine herbicides are amongst the most widely used pesticides in agriculture. These herbicides have been shown to display a slow rate of natural degradation, and pollute the soil, sediments and groundwater [106, 107]. Removal of herbicides from soil is mostly dependent on the catabolic capacity of the soil microflora, where microbial consortia performing the full degradative pathway could ensure that neither triazines nor its intermediates remain in soil after treatment [108]. Therefore, strains from the *P*. *jessenii* group could have potential applications for the biodegradation of these pollutants. Furthermore, the presence in *P*. *jeseenii* strains of several different enzymes involved in xenobiotics degradation, including 2-nitropropane dioxygenase (related to with plant toxic nitroalkanes [109, 110]), an aliphatic nitrilase that is likely to be involved in the detoxification of xenobiotics [111], and two enzymes from the polychlorinated biphenyl degradation pathway [112], make strains from this group particularly suitable for bioremediation applications.

#### Plant-bacteria interaction

Regarding the PGPR abilities of strains from the *P*. *fluorescens* complex, we have identified the clusters of orthologous CDSs for indole-3-acetic acid (IAA) biosynthesis. IAA is the primary plant hormone auxin that has important roles in plant growth and development [113], although it can also be synthesized by microorganism using different pathways [114]. We have found the tryptophan-2-monooxygenase (IaaM) and an indole-3-acetamide (IAM) hydrolase (IaaH), which produces IAA via the IAM pathway. Both enzymes are present in all the strains from the *P*. *chlororaphis* group and in two strains from the *P*. *koreensis* group (S2 File). Bacterial IAA production has previously been reported in *P*. *chlororaphis* O6 [19] and this cluster has been also identified in both, O6 and 3–84 *P*. *chlororaphis* strains [22]. It has been shown that bacterial IAA can induce root growth, increasing plant mineral uptake and root exudation, which enhances bacterial colonization during microorganism-plant interactions [114]. On the other hand, we also have identified a cluster of orthologous CDSs for the degradation of phenylacetic acid (PAA), an auxin analog, in all the strains from the *P*. *chlororaphis* group, although it is also present in some other strains but absent in all the strains from the *P*. *fluorescens* and *P*. *corrugata* groups. PAA catabolism has been reported in several *Pseudomonas* strains and in *E*. *coli* [22, 115, 116] and it has been suggested that PAA plays a role in plant root interaction with microorganisms [117]. Furthermore, it has been shown that PAA produced by bacteria possesses antimicrobial properties [118]. The presence in *P*. *chlororaphis* strains of both IAA biosynthesis and PAA degradation pathways and therefore the ability of this group of bacteria to modify the plant hormonal balance could be crucial for their interactions and the PGPR activity of the strains from this group.

Finally, we have identified the orthologous CDSs for the biosynthesis of the polyamine spermidine, which consist of a S-adenosylmethonine decarboxylase and a spermidine synthase. Both enzymes are present in all the strains from the *P*. *koreensis* group and in a certain number of strains from the *P*. *mandelii*, *P*. *jessenii* and *P*. *corrugata* groups. Both enzymes are required along with putrescine for the biosynthesis of spermidine [119]. It has been shown that PGPR regulate the rate of uptake of polyamines, such as putrescine and spermidine since their accumulation could retard bacterial growth or produce a bactericidal effect [119, 120]. Spermidine, which is also biosynthetized by plants, has been demonstrated to confer resistance to salinity, drought and cold temperatures via its accumulation in plant roots and shoots [121–123]. This might be related to an enhanced competitive colonization of the roots by these strains in those cases in which plants face abiotic stresses and therefore increase the production of spermidine.

The presence of both IAA and PAA metabolic pathways in all the strains from the *P*. *chlororaphis* group suggests that modification of plant hormone levels could be one of the main mechanisms for plant-bacteria interactions common to all *P*. *chlororaphis* strains. However, other mechanisms, such as biosynthesis of spermidine by *P*. *koreensis* strains might also play an important role in this interaction.

Additionally, we have identified a complete additional chemotaxis system that is present in all the strains from the *P*. *corrugata* group and in a very restricted number of strains from the *P*. *mandelii*, *P*. *jessenii*, *P*. *fluorescens* and *P*. *gessardii* groups (S2 File). The functionality of this system has been demonstrated in *P*. *fluorescens* F113 [124] and could be related to the exploitation of specific niches.

## Conclusions

The results presented here show that the *Pseudomonas fluorescens* complex contains at least eight phylogenetic groups that likely represent eco-physiological groups. Each of these groups are formed by several species, and the use of the 70% DDH standard, either in its physical or digital form, will most likely lead to the description of hundreds of additional species within the complex. On the contrary, the number of eight (nine, considering the unsequenced *P*. *fragi* strains) phylogenetic and functional groups is unlikely to dramatically change in the future. As only a single type strain out from more than fifty described species is sequenced, the taxonomic state of the sequenced strains can not be assessed with certainty. Considering the diminishing costs of bacterial genomes sequencing, type strains should be sequenced and the description of new species should include at least a draft genome sequence. Comparative genomics showed a small core genome for the *P*. *fluorescens* complex and a large pan-genome, composed of more than 30,000 CDSs. These values are in agreement with the ecological and genomic diversity of the group. Finally, the analysis of each group-specific genome and the search for key features revealed congruence between the phylogenomic determination of these groups and their eco-physiology. These analyses have shown that the best strains for biocontrol are likely to correspond to *P*. *corrugata*, *P*. *chlororaphis* and *P*. *protegens* groups, while strains from the *P*. *jessenii* group appear more suited for bioremediation/rhizoremediation applications.

## Materials and Methods

### Datasets

Genomes belonging to the genus *Pseudomonas* were downloaded from the NCBI FTP server (ftp.ncbi.nih.gov) in February, 2015. 16S rDNA, *gyrB*, *rpoD* and *rpoB* gene sequences were retrieved from the genomic annotation for each genome. Genomes in which any of these genes could not be retrieved were excluded, resulting in a total of 451 genomes (S1 Table). Predicted proteomes were downloaded either from NCBI or PATRIC FTP servers (ftp.ncbi.nih.gov, ftp.patric.org) in February, 2015. Partial sequences for the 16S rDNA, *gyrB*, *rpoD* and *rpoB* genes for 107 *Pseudomonas* type strains were retrieved from the PseudoMLSA database (http://www.uib.es/microbiologiaBD/Welcome.php). These strains are described in Mulet *et al*., 2010 [8].

### Phylogeny based on MLSA

The MLSA-based phylogenies of both the *Pseudomonas* genus and the *P*. *fluorescens* complex followed the same method: the sequences of *gyrB*, *rpoD* and *rpoB* housekeeping genes along with the 16S rDNA gene sequence were retrieved from the genomic annotation, if available, and by performing BLASTN [125] on the genomic sequence if otherwise. Genes for 107 type strains were retrieved from the PseudoMLSA database. Genes were aligned using Clustal Omega [126]. Resulting alignments were cut and concatenated as described by Mulet *et al*., 2010 [8] using self-written Python scripts. A maximum-likelihood (ML) analysis was performed with the concatenated sequences, using the Timura-Nei model, 1,000 bootstrap replicates and the *P*. *aeruginosa* type strain as outgroup. The tree was obtained, visualized and exported using the MEGA software (v6.06) [127].

### Whole-genome phylogenies

Four different methods were used for the reconstruction of whole-genome phylogenies. In any case, *P*. *aeruginosa* PAO1 was used as outgroup. All trees were obtained, visualized and exported using the MEGA software (v6.06).

#### Composition vector

The predicted proteomes of the 93 sequenced *Pseudomonas fluorescens* complex strains were used to build a phylogenomic tree by a composition vector approach using the CVTree software [45] under a k-mer value of 6. The obtained distance matrix was used to build the phylogenetic tree via the neighbor-joining method (NJ) [128].

#### Specific context-based nucleotide variation

Genomes of the 93 *Pseudomonas* strains were used as input for the Co-phylog software [46], with a structure setting of C_9,9_ O_1_. The obtained distance matrix was used to reconstruct a phylogenetic tree with NJ using the *Neighbor* program contained in PHYLIP [129].

#### Average Nucleotide Identity (ANI)

Genome-to-genome ANI [38] calculations for the 93 *Pseudomonas* genomes with BLAST [130] algorithm implementation were calculated using Python scripts and further converted and parsed into a distance matrix using the formula: 100 –ANIb % similarity. This matrix was then used to construct a phylogenomic tree using the NJ method.

#### Genome BLAST Distance Phylogeny (GBDP)

GBDP [36] was applied to the 93 *Pseudomonas* genomes as previously described [44], at both the nucleotide and amnoacid level and, whole-genome phylogenies were reconstructed from the resulting sets of intergenomic distances (genome-to-genome distances, GGDs). Pseudo-bootstrap [44] branch support values were obtained from 100 replicates. The trees were inferred using FastME v2.07 with TBR postprocessing [131].

### ANI, TETRA and digital DDH calculations

The analysis of sequences for the determination of the relatedness of the 93 *Pseudomonas* strains according to the BLAST-implemented Average Nucleotide Identity (ANIb) and tetranucleotide frequency correlation coefficients (TETRA) were assessed with a python script downloaded from Github (https://github.com/ctSkennerton/scriptShed/blob/master/calculate_ani.py) in conjunction with BLAST for ANI calculations. TETRA was used as an alignment-free genomic similarity index, as oligonucleotide frequencies are carrying a species-specific signal [132]. Digital DDH estimates (dDDH) were calculated with the GGDC 2.0 web service (freely available under http://ggdc.dsmz.de) with the recommended settings [36]. The obtained data are shown in S2 Table.

Collector’s curves based on dDDH and ANIb were assessed by using R [133] scripts to calculate the number of clusters at subspecies, species and groups for dDDH or species and groups for ANIb (according to OPTSIL clusters) depending on sequentially added strains from a total of 200 random replicates.

### Assessment of clusters at distinct levels

Based on the established species delimitation thresholds regarding dDDH [36], ANIb [38] and TETRA [38], the clustering program OPTSIL version 1.5 [134] was applied to identify clusters of species rank. OPTSIL creates a non-hierarchical clustering from a distance threshold *T* and an specific *F* value between zero and one that denotes the fraction of links required for cluster fusion (e.g., *F* values of 0, 0.5 and 1 represent single-, average- and complete-linkage clustering, respectively). Here, as also proposed by [43], an *F* value of 0.5 was chosen. Since the clustering approach requires distances as input, all ANIb similarity values were converted accordingly (100 –ANIb % similarity). As the GGDC starts with calculating GBDP distances, no extra conversion had to be done.

For dDDH values delivered by the GGDC 2.0, a threshold for the delineation of prokaryotic subspecies is also available [43].

OPTSIL was also used to detect the best clustering parameters *T* and *F* to match the eight phylogenomic groups identified in our analysis. These groups were used as a reference partition and the parameters chosen that yielded the highest MRI [135], i.e. the best agreement of the clustering with the partition given by the eight groups.

We further assessed the general suitability of dDDH, ANIb and TETRA to circumscribe species in the *Pseudomonas* dataset with the “clustering consistency” criterion [43]. The higher the mean consistency (across all clusters) at a given threshold *T*, (i) the fewer genome pairs are assigned to the *same* cluster despite their distance being > *T* and (ii) the fewer genome pairs are assigned to *distinct* clusters despite their distance being ≤ *T*. Inconsistencies in species delimitation can generally arise from the use of pairwise distance or similarity thresholds, if the underlying data deviate from a molecular clock (i.e., distances are not ultrametric) [43, 54]. For example, such inconsistencies could result in a specific strain to be assigned to two distinct species at the same time [54]. Since these problems can occur under any given threshold *T*, the ones for the delineation of species, subspecies, and the eight phylogenetic groups within the *Pseudomonas fluorescens* complex, had to be investigated separately. Hence, for all possible genome triplets in our dataset, we assessed whether or not these were consistent at the specific delineation thresholds [54]. This procedure was applied to the dDDH, ANIb and TETRA distance matrices, with the exception that subspecies thresholds are neither established for ANIb nor for TETRA.

### Genome fractions

Identification of orthologous CDSs for each group within the *P*. *fluorescens* complex was conducted comparing all-against-all strains using BLASTP [130] and processed by the OrthoMCL v4 pipeline, using default settings, alignment coverage cut-off 50% and an e-value of 1e-5 [136]. The data were stored in a relational database to further filter results with own Python scripts and SQL queries to retrieve the number of total CDSs in the core genome, strain-specific genome and group-specific genome. To study fluctuations in the number of orthologous sequences within core, strain-specific and pan-genome, R and SQL scripts were implemented to randomly sample strains up to 200 replicates.

## Supporting Information

S1 Fig *Pseudomonas* genus MLSA. MLSA based on partial sequences of 16S rDNA, *gyrB*, *rpoD* and *rpoB* genes from 451 sequenced genomes and 107 type strains (bold), ML method and Tamura-Nei model. *C*. *japonicus* Ueda 107 was used as outgroup. Only bootstrap values above 75% over 1000 replicates are shown. Bold and ^T^ indicates type strain.
(PDF)

S2 Fig Phylogeny of *P*. *fluorescens* complex strains by a context-based nucleotide variation method (Co-phylog). Phylogenomic tree was generated using Co-phylog software [45] with a structure of C_9,9_ O_1_ to build a distance matrix, which was then used to build the phylogenomic tree using the *Neighbor* program found in PHYLIP [126], NJ method and Jukes-Cantor model. *P*. *aeruginosa* PAO1 was used as outgroup. Strains are colored according to the
(PDF)

S3 Fig Phylogeny of *P*. *fluorescens* complex strains based on composition vector approach (CVTree) and GBDP algorithm with amino acid information. (A) Phylogeny is based on all pairwise intergenomic distances between the proteomes as calculated by the latest GBDP version [35] and inferred using FastME v2.07 with TBR postprocessing [128]. Numbers above branches are greedy-with-trimming pseudo-bootstrap [43] support values from 100 replicates and only bootstrap values above 50% are shown. (B) Phylogeny based on a composition vector approach, assessed using CVTree software [44] with a k-mer setting of 6, neighbor-joining (NJ) method and Jukes-Cantor model. *P*. *aeruginosa* PAO1 was used as outgroup. Strains are colored according to the groups established in this work.
(PDF)

S1 File Visualization of both cluster and triplet consistency for the dDDH and ANIb datasets. (PDF)

S2 File Presence of the selected proteins within the clusters of orthologous CDSs for the 93 *P*. *fluorescens* complex strains. Colored boxes indicate the presence of a protein within a strain according to the assigned group color used in this work. White boxes indicate the absence of a protein within a strain.
(XLSX)

S1 Table General genomic features of the *Pseudomonas* sequenced strains used in this work. Retrieved on February, 2015.
(PDF)

S2 Table Affiliation of the 93 *Pseudomonas* strains to clusters defined under various important distance thresholds. The coloring schema represents the colors as established in this work for the eight phylogenomic groups.
(ODS)

S3 Table Reciprocal ANIb, TETRA and dDDH calculations between all the strains of the *P*. *fluorescens* complex. Values for the reciprocal ANIb, TETRA and dDDH calculations between all the strains of the *P*. *fluorescens* complex used in this work.
(XLSX)

S4 Table OPTSIL clustering results of dDDH and ANIb datasets against the reference partition. (ODS)
