# Correction: Genomic and Genetic Diversity within the *Pseudomonas fluorescens* Complex

**DOI:** 10.1371/journal.pone.0153733

**Published:** 2016-04-11

**Authors:** Daniel Garrido-Sanz, Jan P. Meier-Kolthoff, Markus Göker, Marta Martín, Rafael Rivilla, Miguel Redondo-Nieto

There are errors in the names of type strains appearing in Figs 1 and 2 and S1 Fig. *P*. *aureofaciens* LMG 2145 should be *P*. *aureofaciens* 1245. *P*. *amygdali* LMG1384 should be *P*. *amygdali* 13184. *P*. *resinovorans* LMG 2774 should be *P*. *resinovorans* LMG 2274. *P*. *mandelii* LMG 2210 should be *P*. *mandelii* LMG 21607. *P*. *luteola* LMG 21607 should be *P*. *luteola* LMG 7041. Please see the corrected Figs 1 and 2 and S1 Fig here.

**Fig 1:**
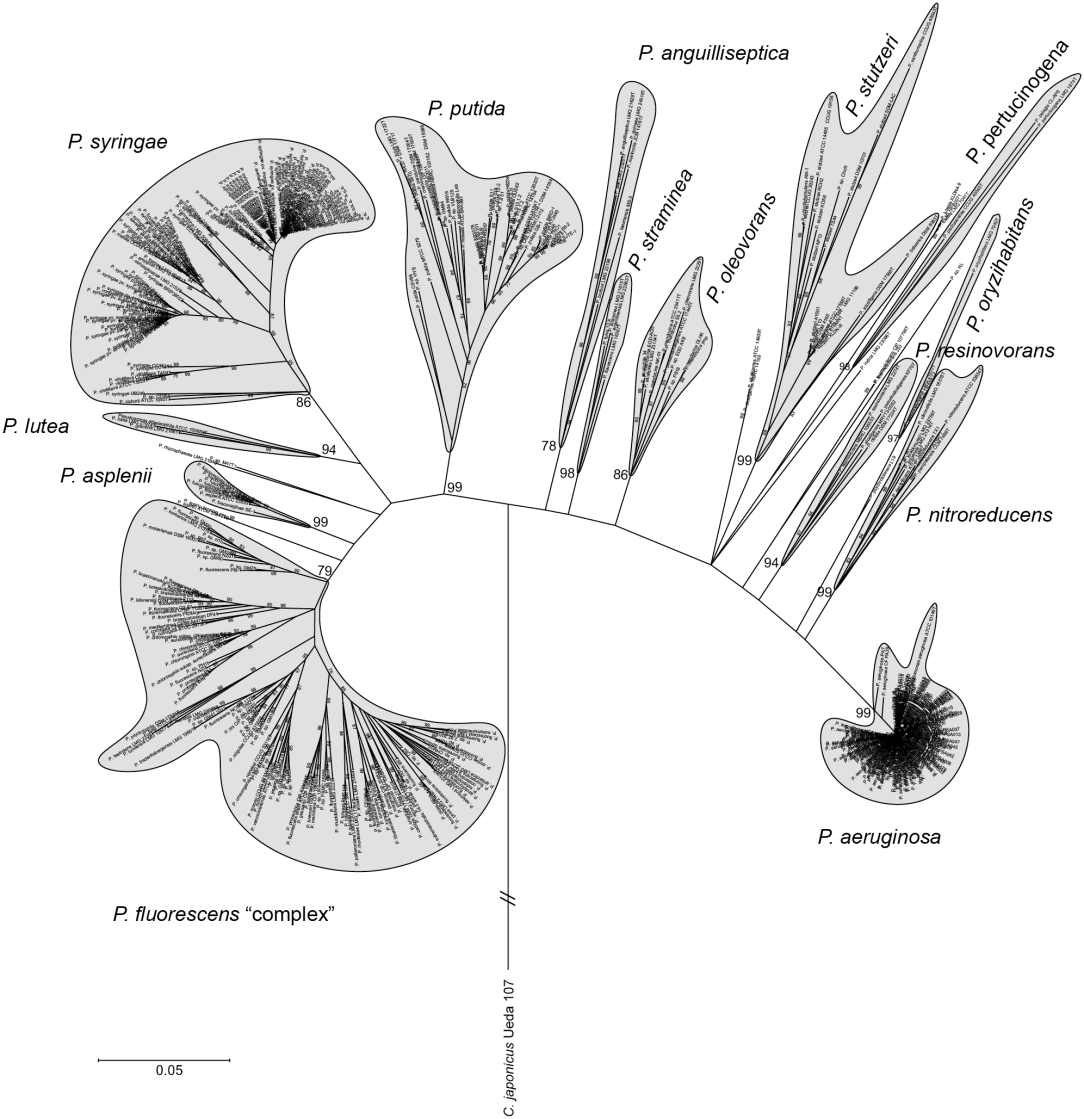
Phylogeny of the Pseudomonas genus inferred by MLSA. Phylogenetic tree of 451 *Pseudomonas* strains along with 107 type strains based on the concatenated partial sequences of the 16S rDNA, *gyrB*, *rpoD* and *rpoB*, ML method, Tamura-Nei. Only bootstrap values above 75% (from 1,000 replicates) are shown. *Cellvibrio japonicum* Ueda 107 was used as outgroup. Details are found in S1 Fig.

**Fig 2:**
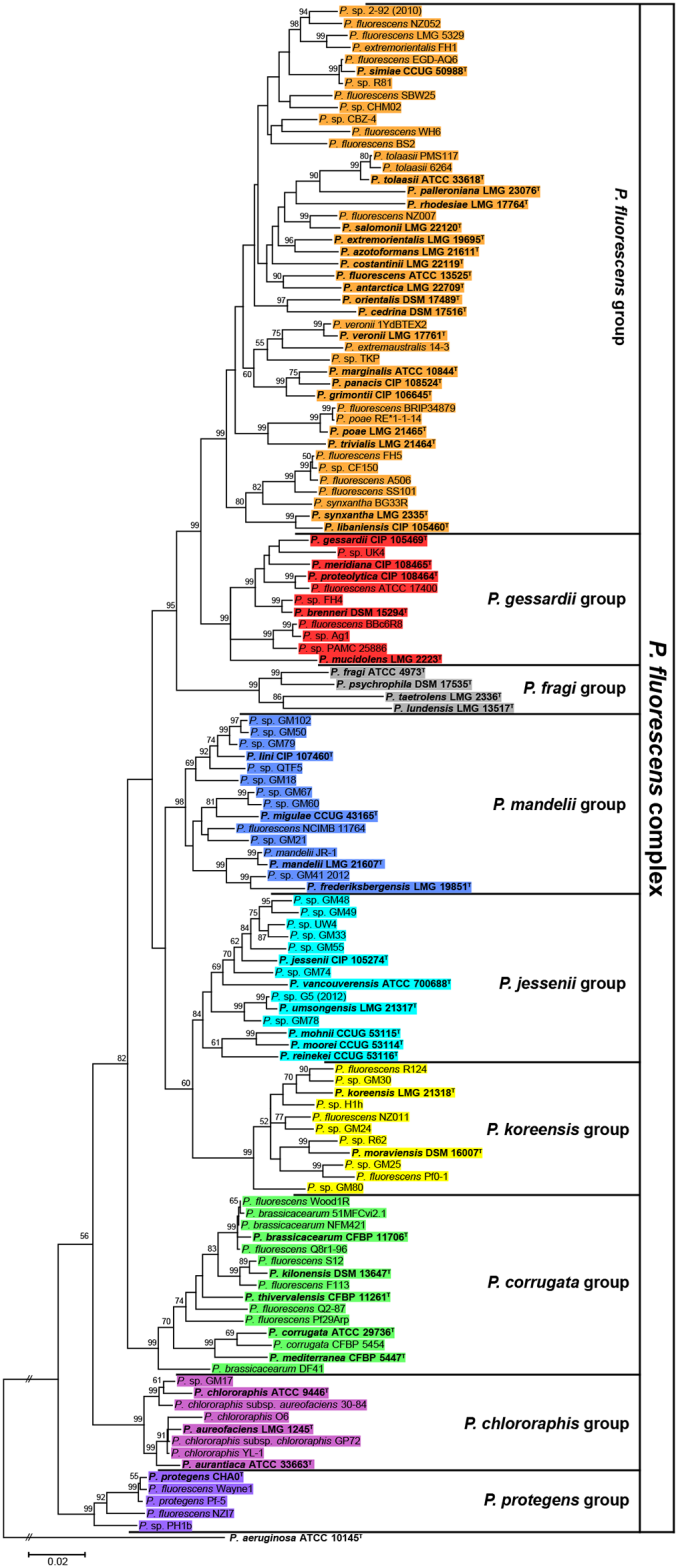
Phylogeny of the P. fluorescens complex inferred by MLSA. Phylogenetic tree of 127 sequenced and type strains belonging to the *P*. *fluorescens* complex based on the concatenated partial sequences of the 16S rDNA, *gyrB*, *rpoD* and *rpoB* genes, the ML method and the Tamura-Nei model. Only bootstrap values above 50% (from 1,000 replicates) are shown. The *P*. *aeruginosa* type strain was used as outgroup. Bold text as well as superscript ^T^ indicates type strains. Strains are colored according to the groups established in this work.

## Supporting Information

S1 Fig Pseudomonas genus MLSA. MLSA based on partial sequences of 16S rDNA, *gyrB*, *rpoD* and *rpoB* genes from 451 sequenced genomes and 107 type strains (bold), ML method and Tamura-Nei model. *C*. *japonicus* Ueda 107 was used as outgroup. Only bootstrap values above 75% over 1000 replicates are shown. Bold and ^T^ indicates type strain.
(PDF)
