# Supplementary figures and images for: Genomic and Genetic Diversity within the Pseudomonas fluorescens Complex
Source: PLoS One. 2016 Feb 25;11(2):e0150183. doi: 10.1371/journal.pone.0150183 (PMC4767706; doi:10.1371/journal.pone.0150183)

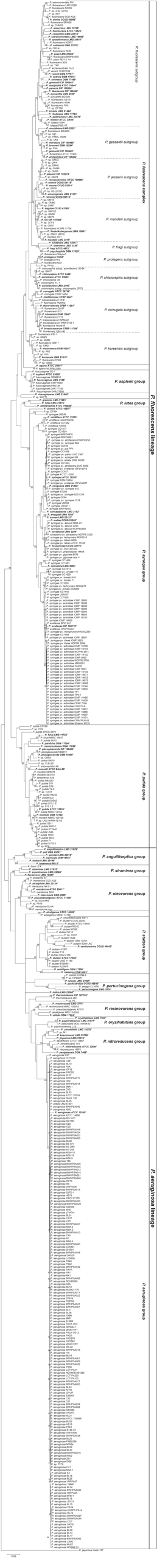

Supplement: S1 Fig — MLSA based on partial sequences of 16S rDNA, gyrB, rpoD and rpoB genes from 451 sequenced genomes and 107 type strains (bold), ML method and Tamura-Nei model. C. japonicus Ueda 107 was used as outgroup. Only bootstrap values above 75% over 1000 replicates are shown. Bold and T indicates type strain. (PDF) [file pone.0150183.s001.pdf]

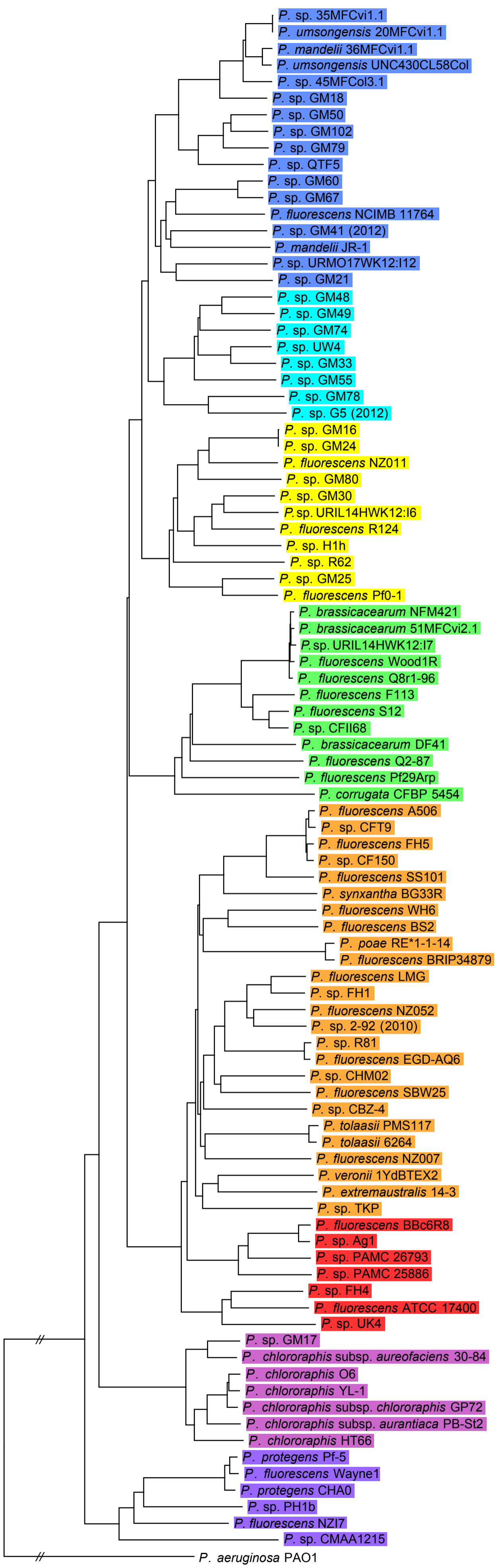

0.05

Supplement: S2 Fig — Phylogenomic tree was generated using Co-phylog software [45] with a structure of C9,9 O1 to build a distance matrix, which was then used to build the phylogenomic tree using the Neighbor program found in PHYLIP [126], NJ method and Jukes-Cantor model. P. aeruginosa PAO1 was used as outgroup. Strains are colored according to the (PDF) [file pone.0150183.s002.pdf]

A

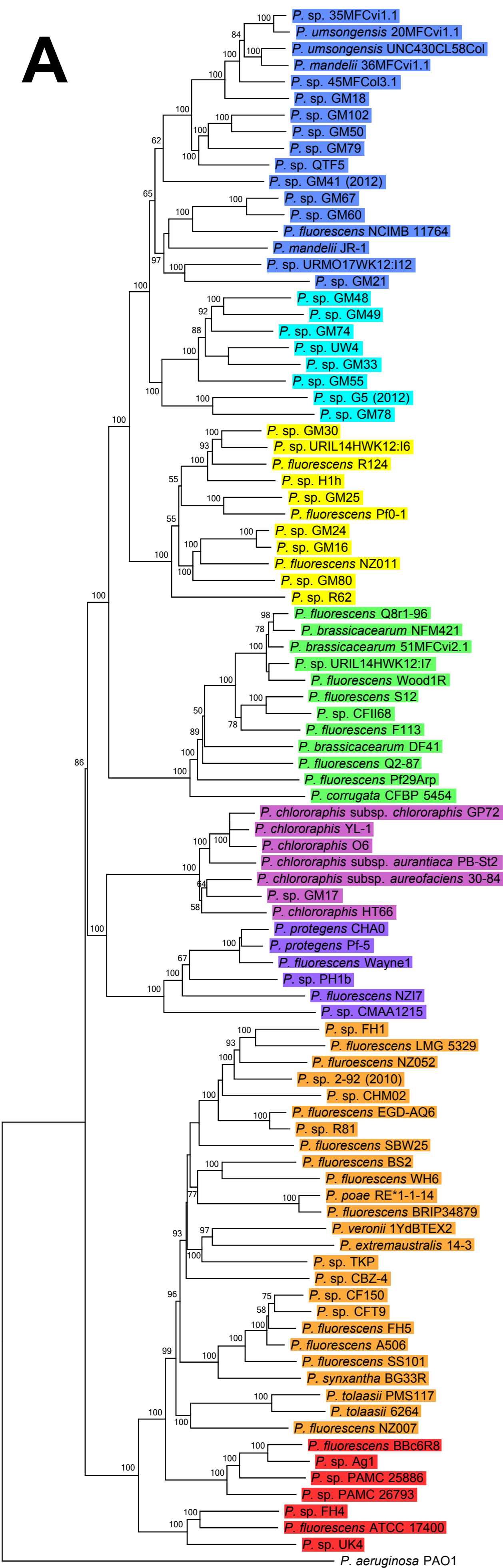

B

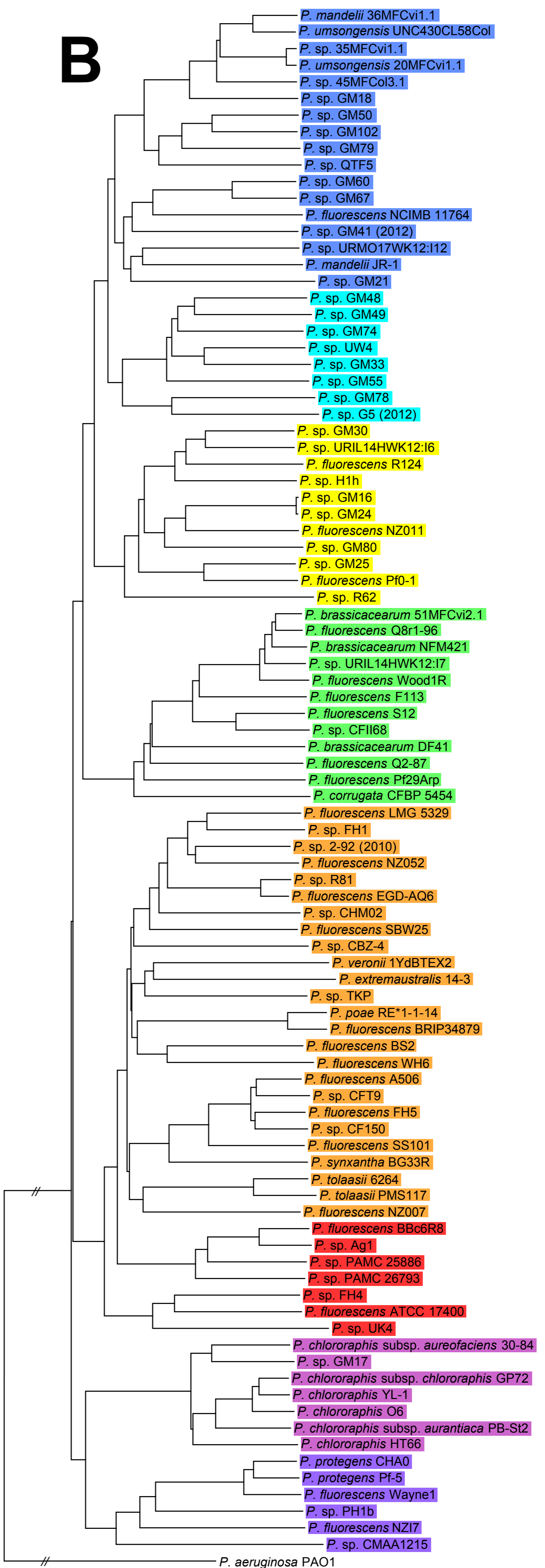

Supplement: S3 Fig — (A) Phylogeny is based on all pairwise intergenomic distances between the proteomes as calculated by the latest GBDP version [35] and inferred using FastME v2.07 with TBR postprocessing [128]. Numbers above branches are greedy-with-trimming pseudo-bootstrap [43] support values from 100 replicates and only bootstrap values above 50% are shown. (B) Phylogeny based on a composition vector approach, assessed using CVTree software [44] with a k-mer setting of 6, neighbor-joining (NJ) method and Jukes-Cantor model. P. aeruginosa PAO1 was used as outgroup. Strains are colored according to the groups established in this work. (PDF) [file pone.0150183.s003.pdf]
