# Supplementary material for: Genomic and Genetic Diversity within the Pseudomonas fluorescens Complex
Source: PLoS One. 2016 Feb 25;11(2):e0150183. doi: 10.1371/journal.pone.0150183 (PMC4767706; doi:10.1371/journal.pone.0150183)
Supplement: S1 File — (PDF) [file pone.0150183.s004.pdf]

We investigated the clustering consistency over the complete ranges of *T* and *F*. The results regarding dDDH and ANIb are shown in the following figures. At the relevant thresholds established for the delimitation of subspecies (0.02454), species (0.03611) and the eight phylogenetic groups (0.13292), dDDH yielded mean consistency values of 1, 1 and 0.989, respectively (rounded to three decimal places). ANIb yielded slightly lower values, that is, with respect to the distance thresholds for species (0.05) and the phylogenomic groups (0.152), a mean clustering consistency of 0.997 and 0.952, respectively. All of the previously reported highest mean consistency values were found under an *F* measure of 0.5 (i.e., average-linkage clustering).

The triplet consistency was also assessed regarding dDDH and ANIb and is depicted below the clustering consistency in the following plots. For dDDH and with respect to the species and subspecies thresholds all triplets were consistent and only the threshold for the eight phylogenetic groups yielded a reduced triplet consistency of about 0.825. Regarding ANIb, the triplet consistency was significantly lower at both the thresholds for species delineation and the eight phylogenetic groups: 0.666 and 0.755, respectively.

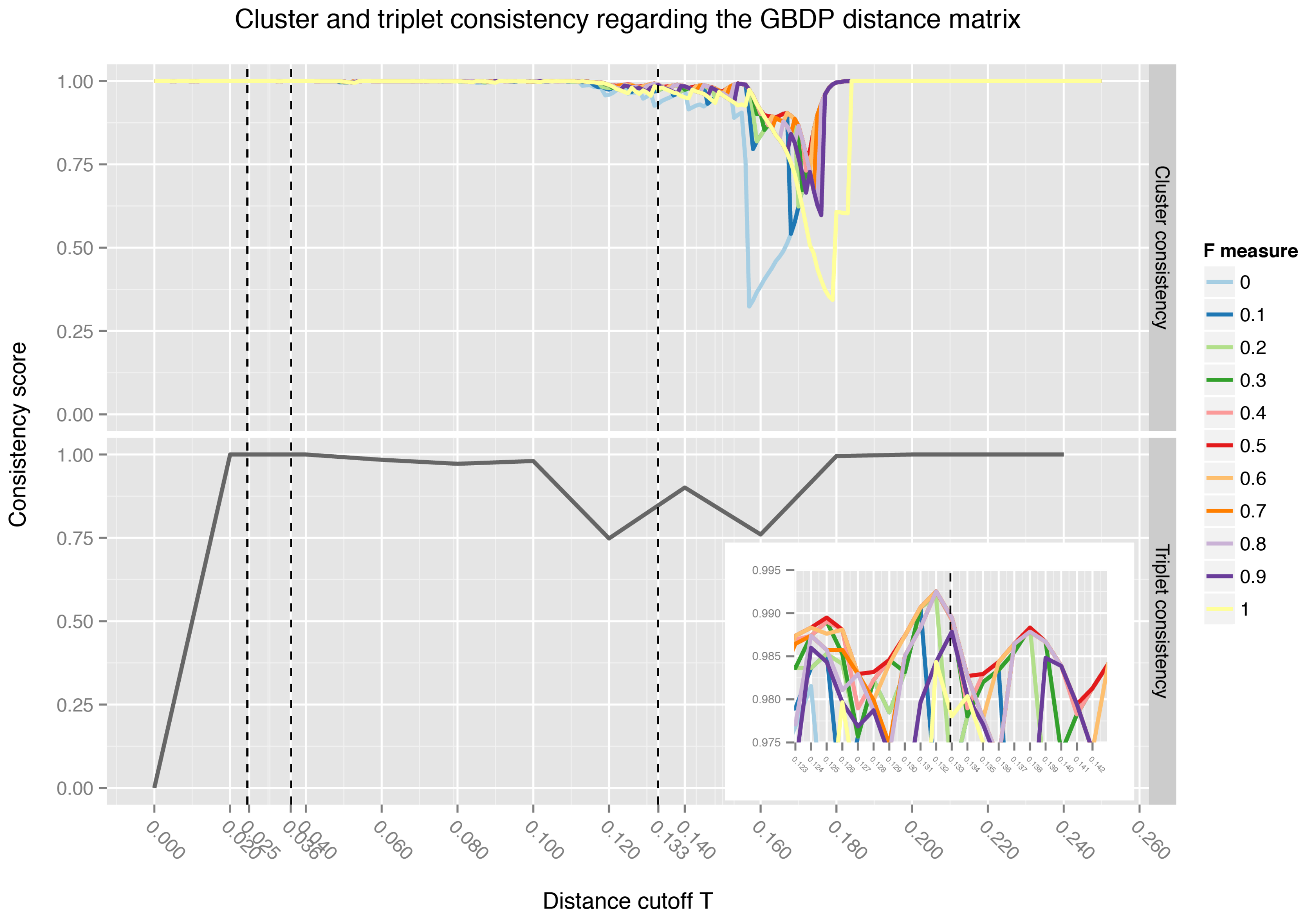

Cluster and triplet consistency regarding the GBDP distance matrix
Consistency score
Distance cutoff T
Cluster consistency
Triplet consistency
F measure
0
0.1
0.2
0.3
0.4
0.5
0.6
0.7
0.8
0.9
1
1.00
0.75
0.50
0.25
0.00
0.000
0.020
0.025
0.036
0.040
0.060
0.080
0.100
0.120
0.133
0.140
0.160
0.180
0.200
0.220
0.240
0.260
0.995
0.990
0.985
0.980
0.975

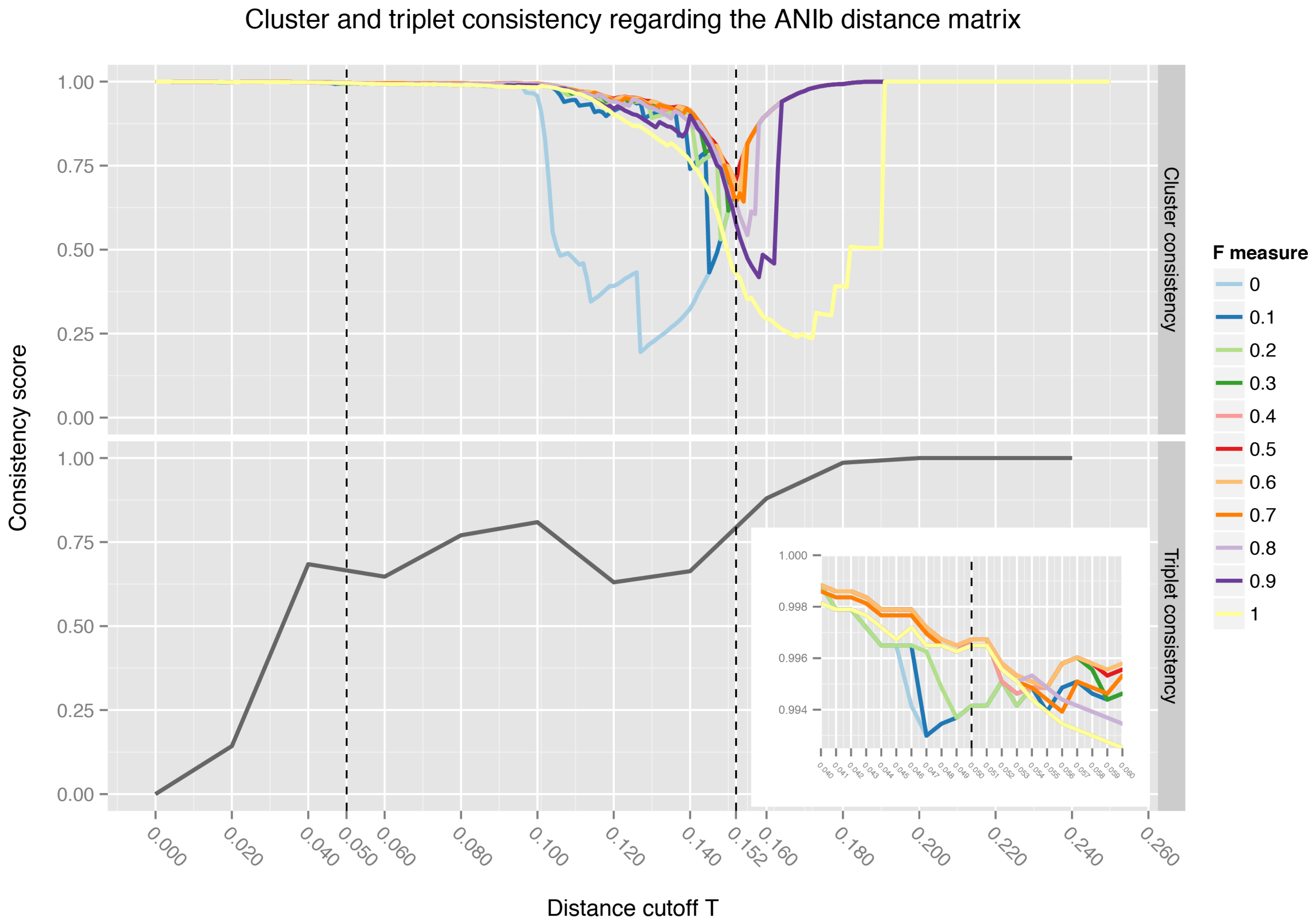

Cluster and triplet consistency regarding the ANIb distance matrix
Consistency score
1.00
0.75
0.50
0.25
0.00
Cluster consistency
Triplet consistency
1.000
0.998
0.996
0.994
0.040
0.041
0.042
0.043
0.044
0.045
0.046
0.047
0.048
0.049
0.050
0.051
0.052
0.053
0.054
0.055
0.056
0.057
0.058
0.059
0.060
0.000
0.020
0.040
0.050
0.060
0.080
0.100
0.120
0.140
0.152
0.160
0.180
0.200
0.220
0.240
0.260
Distance cutoff T
F measure
0
0.1
0.2
0.3
0.4
0.5
0.6
0.7
0.8
0.9
1
