# Supplementary material for: Genomic and Genetic Diversity within the Pseudomonas fluorescens Complex
Source: PLoS One. 2016 Feb 25;11(2):e0150183. doi: 10.1371/journal.pone.0150183 (PMC4767706; doi:10.1371/journal.pone.0150183)
Supplement: S1 Table — Retrieved on February, 2015. (PDF) [file pone.0150183.s006.pdf]

Strain	Assembly	Size (Mb)	GC%	Scaffolds	Genes	Proteins	Level
P. aeruginosa 18A	GCA_000341565.1	6.09	66.5	179	5755	5681	Contig
P. aeruginosa 19660	GCA_000481765.1	6.75	66.2	11	6271	6193	Scaffold
P. aeruginosa 19BR	GCA_000223945.2	6.74	66.1	1	6258	6145	Complete Genome
P. aeruginosa 213BR	GCA_000223965.2	6.72	66.1	1	6232	6126	Complete Genome
P. aeruginosa 39016	GCA_000148745.1	6.87	65.5	1	6429	6346	Chromosome
P. aeruginosa 6077	GCA_000481745.1	7.00	66.0	13	6518	6431	Scaffold
P. aeruginosa 62	GCA_000482025.1	6.55	66.3	5	6039	5960	Scaffold
P. aeruginosa 9BR	GCA_000223925.2	6.85	66.1	2	6387	6279	Contig
P. aeruginosa AH16	GCA_000287875.1	6.77	66.1	130	6331	6180	Contig
P. aeruginosa ATCC 14886	GCA_000297275.1	6.38	66.4	211	5840	5715	Contig
P. aeruginosa ATCC 25324	GCA_000297295.1	7.10	65.9	1023	7042	6785	Contig
P. aeruginosa ATCC 700888	GCA_000297315.1	6.79	66.1	600	6514	6370	Contig
P. aeruginosa B136-33	GCA_000359505.1	6.42	66.4	1	5876	5779	Complete Genome
P. aeruginosa B3-1811	GCA_000455545.1	6.68	66.2	341	6343	6245	Contig
P. aeruginosa B3-208	GCA_000455405.1	6.73	66.2	418	6445	6356	Contig
P. aeruginosa B3-20M	GCA_000455505.1	6.73	66.2	338	6387	6264	Contig
P. aeruginosa B3-CFI	GCA_000455425.1	6.75	66.2	314	6406	6279	Contig
P. aeruginosa BK1	GCA_000572265.1	6.45	66.3	163	-	-	Contig
P. aeruginosa BL01	GCA_000481125.1	6.44	66.4	4	5903	5831	Scaffold
P. aeruginosa BL02	GCA_000481105.1	6.91	66.1	6	6454	6372	Scaffold
P. aeruginosa BL03	GCA_000481085.1	7.06	66.1	7	6521	6444	Scaffold
P. aeruginosa BL04	GCA_000481065.1	7.33	65.9	11	6877	6793	Scaffold
P. aeruginosa BL05	GCA_000481045.1	6.38	66.5	3	5852	5784	Scaffold
P. aeruginosa BL06	GCA_000481025.1	7.00	65.9	13	6505	6435	Scaffold
P. aeruginosa BL07	GCA_000481005.1	6.56	66.3	9	6017	5940	Scaffold
P. aeruginosa BL08	GCA_000480985.1	6.98	65.9	9	6492	6415	Scaffold
P. aeruginosa BL09	GCA_000480965.1	6.93	66.0	11	6364	6281	Scaffold
P. aeruginosa BL10	GCA_000480945.1	6.50	66.3	3	5983	5909	Scaffold
P. aeruginosa BL11	GCA_000480925.1	7.08	65.9	8	6533	6456	Scaffold
P. aeruginosa BL12	GCA_000480905.1	7.47	65.6	19	7003	6923	Scaffold
P. aeruginosa BL13	GCA_000480885.1	7.29	65.9	4	6693	6618	Scaffold
P. aeruginosa BL14	GCA_000480865.1	7.11	65.9	8	6535	6455	Scaffold
P. aeruginosa BL15	GCA_000480845.1	6.31	66.5	9	5790	5710	Scaffold
P. aeruginosa BL16	GCA_000480825.1	6.81	66.0	10	6310	6230	Scaffold
P. aeruginosa BL17	GCA_000480805.1	6.88	66.0	3	6376	6299	Scaffold
P. aeruginosa BL18	GCA_000480785.1	6.51	66.4	3	6000	5930	Scaffold
P. aeruginosa BL19	GCA_000480765.1	6.38	66.4	5	5860	5783	Scaffold
P. aeruginosa BL20	GCA_000480745.1	6.74	66.2	18	6154	6072	Scaffold
P. aeruginosa BL21	GCA_000480725.1	6.84	66.1	3	6325	6247	Scaffold
P. aeruginosa BL22	GCA_000480705.1	7.00	66.1	12	6447	6366	Scaffold
P. aeruginosa BL23	GCA_000480685.1	7.15	65.7	16	6687	6606	Scaffold
P. aeruginosa BL24	GCA_000480665.1	6.96	66.1	8	6410	6336	Scaffold
P. aeruginosa BL25	GCA_000480645.1	6.57	66.3	7	6047	5965	Scaffold
P. aeruginosa BWHPSA001	GCA_000481685.1	6.49	66.4	8	5953	5880	Scaffold
P. aeruginosa BWHPSA002	GCA_000481665.1	6.94	66.1	11	6420	6339	Scaffold
P. aeruginosa BWHPSA003	GCA_000481645.1	6.89	66.0	13	6328	6250	Scaffold

P. aeruginosa BWHPSA004	GCA_000481625.1	6.31	66.5	3	5788	5712	Scaffold
P. aeruginosa BWHPSA005	GCA_000481605.1	6.79	66.1	4	6307	6230	Scaffold
P. aeruginosa BWHPSA006	GCA_000481585.1	6.96	66.2	8	6418	6340	Scaffold
P. aeruginosa BWHPSA007	GCA_000481565.1	6.83	66.2	6	6318	6246	Scaffold
P. aeruginosa BWHPSA008	GCA_000481545.1	6.52	66.3	5	6033	5960	Scaffold
P. aeruginosa BWHPSA009	GCA_000481525.1	6.47	66.4	18	5954	5879	Scaffold
P. aeruginosa BWHPSA010	GCA_000481505.1	6.86	66.1	21	6482	6393	Scaffold
P. aeruginosa BWHPSA011	GCA_000481485.1	7.07	65.8	11	6607	6531	Scaffold
P. aeruginosa BWHPSA012	GCA_000481465.1	6.49	66.3	4	5982	5905	Scaffold
P. aeruginosa BWHPSA013	GCA_000481445.1	6.70	66.2	2	6190	6113	Scaffold
P. aeruginosa BWHPSA014	GCA_000481425.1	6.29	66.5	2	5785	5707	Scaffold
P. aeruginosa BWHPSA015	GCA_000481405.1	6.43	66.5	5	5907	5833	Scaffold
P. aeruginosa BWHPSA016	GCA_000481385.1	6.48	66.4	3	6020	5945	Scaffold
P. aeruginosa BWHPSA017	GCA_000481365.1	6.93	66.0	5	6380	6302	Scaffold
P. aeruginosa BWHPSA018	GCA_000481345.1	6.65	66.2	9	6155	6078	Scaffold
P. aeruginosa BWHPSA019	GCA_000481325.1	6.44	66.4	3	5930	5858	Scaffold
P. aeruginosa BWHPSA020	GCA_000481305.1	6.53	66.3	24	5987	5915	Scaffold
P. aeruginosa BWHPSA021	GCA_000481285.1	6.48	66.3	4	5988	5909	Scaffold
P. aeruginosa BWHPSA022	GCA_000481265.1	6.91	66.2	6	6454	6373	Scaffold
P. aeruginosa BWHPSA023	GCA_000481245.1	6.93	66.1	16	6418	6344	Scaffold
P. aeruginosa BWHPSA024	GCA_000481225.1	6.77	66.1	5	6313	6239	Scaffold
P. aeruginosa BWHPSA025	GCA_000481205.1	6.34	66.5	4	5811	5731	Scaffold
P. aeruginosa BWHPSA026	GCA_000481185.1	6.80	66.0	11	6313	6232	Scaffold
P. aeruginosa BWHPSA027	GCA_000481165.1	6.88	66.0	8	6382	6301	Scaffold
P. aeruginosa BWHPSA028	GCA_000481145.1	7.48	65.3	14	7013	6934	Scaffold
P. aeruginosa BWHPSA037	GCA_000520455.1	7.22	65.6	29	6785	6699	Scaffold
P. aeruginosa BWHPSA038	GCA_000520435.1	6.65	66.3	12	6128	6052	Scaffold
P. aeruginosa BWHPSA039	GCA_000520415.1	6.98	66.0	10	6422	6344	Scaffold
P. aeruginosa BWHPSA040	GCA_000520395.1	6.45	66.4	3	5891	5814	Scaffold
P. aeruginosa BWHPSA041	GCA_000520375.1	6.90	65.9	12	6471	6394	Scaffold
P. aeruginosa BWHPSA042	GCA_000520355.1	5.89	66.5	3	5483	5416	Scaffold
P. aeruginosa BWHPSA043	GCA_000520335.1	6.88	66.0	27	6378	6288	Scaffold
P. aeruginosa BWHPSA044	GCA_000520315.1	6.90	66.0	4	6398	6321	Scaffold
P. aeruginosa BWHPSA045	GCA_000520295.1	7.08	65.8	5	6637	6556	Scaffold
P. aeruginosa BWHPSA046	GCA_000520275.1	6.90	66.1	10	6454	6372	Scaffold
P. aeruginosa BWHPSA047	GCA_000520255.1	6.39	66.4	3	5932	5857	Scaffold
P. aeruginosa BWHPSA048	GCA_000520235.1	5.95	66.4	2	5508	5431	Scaffold
P. aeruginosa C20	GCA_000480515.1	6.93	66.2	3	6420	6352	Scaffold
P. aeruginosa C23	GCA_000480495.1	6.90	66.2	3	6423	6350	Scaffold
P. aeruginosa C40	GCA_000480475.1	6.63	66.3	4	6130	6058	Scaffold
P. aeruginosa C41	GCA_000480455.1	6.83	66.2	3	6340	6263	Scaffold
P. aeruginosa C48	GCA_000480435.1	6.41	66.4	6	5875	5799	Scaffold
P. aeruginosa C51	GCA_000480415.1	6.91	66.1	18	6383	6307	Scaffold
P. aeruginosa C52	GCA_000480395.1	7.10	65.9	8	6547	6470	Scaffold
P. aeruginosa c7447m	GCA_000468935.1	6.26	66.5	1	5731	5657	Chromosome with gaps
P. aeruginosa CF_PA39	GCA_000568235.1	6.19	66.4	98	5684	5569	Contig
P. aeruginosa CF127	GCA_000481945.1	7.03	65.9	7	6447	6365	Scaffold

P. aeruginosa CF18	GCA_000481925.1	6.49	66.3	6	6011	5934	Scaffold
P. aeruginosa CF27	GCA_000481905.1	6.54	66.3	5	5966	5895	Scaffold
P. aeruginosa CF5	GCA_000481885.1	6.34	66.6	5	5851	5779	Scaffold
P. aeruginosa CF614	GCA_000480355.1	6.80	66.0	7	6284	6200	Scaffold
P. aeruginosa CI27	GCA_000297335.1	6.77	66.1	160	6178	6034	Contig
P. aeruginosa CIG1	GCA_000295475.1	6.54	66.0	544	6221	6028	Contig
P. aeruginosa DHS01	GCA_000496455.1	6.95	65.8	106	6646	6522	Contig
P. aeruginosa DHS29	GCA_000503175.1	7.11	65.8	267	6807	6681	Contig
P. aeruginosa DK2	GCA_000271365.1	6.40	66.3	1	5920	5805	Complete Genome
P. aeruginosa DQ8	GCA_000283055.1	6.78	66.0	376	6503	6299	Contig
P. aeruginosa E2	GCA_000482005.1	6.41	66.4	10	5858	5781	Scaffold
P. aeruginosa H1l	GCA_000633495.1	6.40	66.4	40	5936	5861	Contig
P. aeruginosa HB13	GCA_000215775.4	6.53	66.2	24	6209	6122	Contig
P. aeruginosa HB15	GCA_000215795.4	6.64	66.2	20	6155	6053	Contig
P. aeruginosa JD331	GCA_000506305.1	6.31	66.5	2377	-	-	Contig
P. aeruginosa JJ692	GCA_000481805.1	6.75	66.1	5	6235	6160	Scaffold
P. aeruginosa LCT-PA102	GCA_000258285.1	6.75	66.2	180	6305	6224	Contig
P. aeruginosa LCT-PA220	GCA_000439855.1	6.85	66.2	82	6320	6232	Scaffold
P. aeruginosa LCT-PA41	GCA_000439875.1	6.85	66.2	88	6326	6236	Scaffold
P. aeruginosa LES431	GCA_000508765.1	6.55	66.3	1	6059	5943	Complete Genome
P. aeruginosa LESB58	GCA_000026645.1	6.60	66.3	1	6132	6025	Complete Genome
P. aeruginosa M18	GCA_000226155.1	6.33	66.5	1	5825	5728	Complete Genome
P. aeruginosa M8A.1	GCA_000480615.1	6.37	66.4	3	5834	5758	Scaffold
P. aeruginosa M8A.2	GCA_000480595.1	6.38	66.4	6	5868	5786	Scaffold
P. aeruginosa M8A.3	GCA_000480575.1	6.37	66.5	4	5833	5756	Scaffold
P. aeruginosa M8A.4	GCA_000480555.1	6.42	66.5	5	5865	5797	Scaffold
P. aeruginosa M9A.1	GCA_000480535.1	6.43	66.4	10	5918	5841	Scaffold
P. aeruginosa MH27	GCA_000513235.1	7.16	65.9	31	6378	6297	Contig
P. aeruginosa MPAO1/P1	GCA_000247435.2	6.26	66.5	140	5858	5791	Contig
P. aeruginosa MPAO1/P2	GCA_000247455.2	6.28	66.5	301	5981	5914	Contig
P. aeruginosa MRW44.1	GCA_000282915.1	6.27	66.5	54	5787	5696	Contig
P. aeruginosa MSH-10	GCA_000407905.1	6.50	66.4	2	5994	5912	Scaffold
P. aeruginosa MSH10	GCA_000481965.1	6.50	66.4	4	5991	5916	Scaffold
P. aeruginosa MSH3	GCA_000481985.1	6.51	66.4	7	6004	5927	Scaffold
P. aeruginosa MTB-1	GCA_000504045.1	6.58	66.2	1	6077	5976	Complete Genome
P. aeruginosa MW3a	GCA_000590905.1	6.67	66.3	240	6256	6176	Contig
P. aeruginosa N002	GCA_000287815.2	6.05	66.8	235	5642	5553	Contig
P. aeruginosa NCAIM B.001380	GCA_000685845.1	6.99	66.1	15	6499	6279	Contig
P. aeruginosa NCGM2.S1	GCA_000284555.1	6.76	66.1	1	6278	6160	Complete Genome
P. aeruginosa NCMG1179	GCA_000291745.1	7.06	66.0	92	6356	6260	Scaffold
P. aeruginosa PA1	GCA_000496605.1	6.53	66.3	1	6055	5953	Complete Genome
P. aeruginosa PA14	GCA_000404265.1	6.58	66.3	4	5994	5919	Scaffold
P. aeruginosa PA1R	GCA_000496645.1	6.31	66.3	1	5863	5760	Complete Genome
P. aeruginosa PA21_ST175	GCA_000342145.1	6.89	66.1	90	6532	6468	Contig
P. aeruginosa PA45	GCA_000359565.1	6.62	66.3	124	6233	6172	Contig
P. aeruginosa PA7	GCA_000017205.1	6.59	66.4	1	6079	5955	Complete Genome
P. aeruginosa PAb1	GCA_000172395.1	6.08	66.8	658	5894	5788	Contig

P. aeruginosa PABL056	GCA_000290555.1	7.28	65.5	391	7131	7030	Scaffold
P. aeruginosa PACS2	GCA_000168335.1	6.49	66.3	1	5989	5904	Complete Genome
P. aeruginosa PAK	GCA_000408865.1	6.41	66.4	1	5888	5808	Scaffold
P. aeruginosa PAO1	GCA_000006765.1	6.26	66.6	1	5697	5572	Complete Genome
P. aeruginosa PAO1-VE13	GCA_000484545.1	6.27	66.6	1	5752	5671	Chromosome with gaps
P. aeruginosa PAO1-VE2	GCA_000484495.1	6.27	66.6	1	5751	5670	Chromosome with gaps
P. aeruginosa PAO579	GCA_000296325.1	6.02	66.5	16	5600	5522	Contig
P. aeruginosa PAO581	GCA_000468555.1	6.04	66.5	1	5577	5500	Chromosome with gaps
P. aeruginosa PGPR2	GCA_000412735.1	6.77	66.0	198	6250	5520	Contig
P. aeruginosa PS42	GCA_000520195.1	7.53	65.5	19	7049	6971	Scaffold
P. aeruginosa PS50	GCA_000520175.1	6.90	66.0	4	6336	6263	Scaffold
P. aeruginosa RB	GCA_000647935.2	6.20	66.6	78	5740	5661	Scaffold
P. aeruginosa RB-48	GCA_000568115.1	6.18	66.5	183	5792	5720	Contig
P. aeruginosa RP73	GCA_000414035.1	6.34	66.5	1	5856	5737	Complete Genome
P. aeruginosa S35004	GCA_000481725.1	7.00	66.1	9	6429	6351	Scaffold
P. aeruginosa S54485	GCA_000481825.1	7.01	66.0	8	6586	6509	Scaffold
P. aeruginosa SCV20265	GCA_000510305.1	6.73	66.3	1	6261	6159	Complete Genome
P. aeruginosa SG17M	GCA_000568215.1	6.88	66.0	17	6345	6258	Contig
P. aeruginosa SJTD-1	GCA_000271985.1	6.07	66.8	104	5613	5530	Contig
P. aeruginosa str. Stone 130	GCA_000478465.2	7.50	65.4	6	7112	7038	Scaffold
P. aeruginosa U2504	GCA_000481785.1	7.10	65.9	11	6658	6581	Scaffold
P. aeruginosa UCBPP-PA14	GCA_000014625.1	6.54	66.3	1	5980	5883	Complete Genome
P. aeruginosa UDL	GCA_000481845.1	6.31	66.5	2	5788	5711	Scaffold
P. aeruginosa VRFPA05	GCA_000496325.1	7.05	65.8	170	6667	6155	Contig
P. aeruginosa VRFPA06	GCA_000567865.1	6.98	65.8	277	6650	6277	Contig
P. aeruginosa VRFPA07	GCA_000506805.1	7.18	65.9	140	6915	6764	Contig
P. aeruginosa VRFPA08	GCA_000506885.1	7.04	66.1	197	6794	6661	Contig
P. aeruginosa X13273	GCA_000481705.1	7.04	66.0	15	6594	6518	Scaffold
P. aeruginosa X24509	GCA_000481865.1	6.45	66.3	5	5946	5870	Scaffold
P. aeruginosa XMG	GCA_000265035.1	6.45	66.4	225	6023	5946	Contig
P. aeruginosa Z61	GCA_000520215.1	6.56	66.3	5	5992	5906	Scaffold
P. agarici NCPPB 2289	GCA_000280785.1	5.51	59.1	119	4919	4945	Scaffold
P. alcaligenes MRY13-0052	GCA_000474255.1	6.88	65.8	237	6222	6112	Contig
P. alcaligenes NBRC 14159	GCA_000467105.1	4.82	64.8	122	4588	4531	Contig
P. alcaliphila 34	GCA_000319815.1	5.43	62.6	18	4998	4901	Contig
P. avellanae BPIC 631	GCA_000302915.1	6.63	58.7	297	4864	4757	Scaffold
P. avellanae CRAFRUec1	GCA_000441975.1	5.74	58.9	547	5202	5016	Contig
P. brassicacearum 51MFCVI2.1[a]	GCA_000510785.1	6.57	61.0	49	5846	5768	Scaffold
P. brassicacearum DF41[a]	GCA_000585995.1	6.65	60.5	1	5745	5584	Complete Genome
P. brassicacearum subsp. *brassicacearum* NFM421[a]	GCA_000194805.1	6.84	60.8	1	6056	5931	Complete Genome
P. chloritidismutans AW-1	GCA_000495915.1	5.06	62.5	77	4870	4767	Contig
P. chlororaphis HT66[a]	GCA_000597925.1	7.30	62.6	50	6504	6403	Contig
P. chlororaphis O6[a]	GCA_000264555.1	6.98	62.9	1	6248	6140	Chromosome
P. chlororaphis subsp. *aurantiaca* PB-St2[a]	GCA_000506385.1	6.59	63.2	23	5975	5860	Contig
P. chlororaphis subsp. *aureofaciens* 30-84[a]	GCA_000281915.1	6.67	62.9	1	5903	5787	Chromosome
P. chlororaphis subsp. *chlororaphis* GP72[a]	GCA_000237045.2	6.63	63.1	347	6074	5997	Contig

P. chlororaphis YL-1[a]	GCA_000512485.1	6.80	63.1	82	6125	6056	Scaffold
P. corrugata CFBP 5454[a]	GCA_000522485.1	6.19	60.5	157	5471	5395	Contig
P. denitrificans ATCC 13867	GCA_000349845.1	5.70	65.2	1	5090	4990	Complete Genome
P. entomophila L48	GCA_000026105.1	5.89	64.2	1	5151	5032	Complete Genome
P. extremaustralis 14-3[a]	GCA_000242115.2	6.59	60.7	135	5989	5870	Contig
P. fluorescens A506[a]	GCA_000262325.2	6.02	59.9	2	5446	5315	Complete Genome
P. fluorescens ATCC 17400[a]	GCA_000708695.1	6.25	60.5	75	5589	5475	Contig
P. fluorescens BBc6R8[a]	GCA_000297195.2	6.95	61.0	153	6436	6292	Contig
P. fluorescens BRIP34879[a]	GCA_000334015.1	5.53	60.9	110	4961	4903	Scaffold
P. fluorescens BS2[a]	GCA_000308175.1	6.12	60.6	122	5608	5501	Contig
P. fluorescens EGD-AQ6[a]	GCA_000465595.1	6.09	60.5	58	5665	5593	Contig
P. fluorescens F113[a]	GCA_000237065.1	6.85	60.8	1	6046	5927	Complete Genome
P. fluorescens FH5[a]	GCA_000511155.1	6.18	60.0	88	5664	5598	Contig
P. fluorescens LMG 5329[a]	GCA_000411675.1	6.87	60.5	253	6231	6126	Contig
P. fluorescens NCIMB 11764[a]	GCA_000293885.2	7.02	59.0	1	6404	6297	Chromosome
P. fluorescens NZ007[a]	GCA_000280805.1	6.54	60.0	141	5879	5642	Scaffold
P. fluorescens NZ011[a]	GCA_000276585.1	6.81	58.5	973	5862	5607	Contig
P. fluorescens NZ052[a]	GCA_000275925.1	6.83	60.1	440	6241	6026	Contig
P. fluorescens NZI7[a]	GCA_000275905.1	6.81	63.2	1032	6643	6112	Contig
P. fluorescens Pf0-1[a]	GCA_000012445.1	6.44	60.5	1	5779	5657	Complete Genome
P. fluorescens Pf29Arp[a]	GCA_000346775.1	6.13	60.9	69	5540	5414	Scaffold
P. fluorescens Q2-87[a]	GCA_000281895.1	6.37	60.6	1	5663	5530	Chromosome
P. fluorescens Q8r1-96[a]	GCA_000263695.2	6.60	61.0	1	5817	5713	Chromosome
P. fluorescens R124[a]	GCA_000292795.1	6.30	60.3	2	5609	5499	Chromosome
P. fluorescens S12[a]	GCA_000498415.1	6.37	60.7	332	5642	5560	Contig
P. fluorescens SBW25[a]	GCA_000009225.1	6.72	60.5	1	6042	5929	Complete Genome
P. fluorescens SS101[a]	GCA_000263675.2	6.18	60.0	1	5451	5331	Chromosome
P. fluorescens Wayne1[a]	GCA_000285355.1	6.82	63.4	337	6270	6231	Contig
P. fluorescens WH6[a]	GCA_000166515.1	6.27	60.6	1	5747	5461	Chromosome
P. fluorescens Wood1R[a]	GCA_000285615.1	6.68	60.8	1437	6161	6118	Contig
P. fulva 12-X	GCA_000213805.1	4.92	63.5	1	4499	4404	Complete Genome
P. fulva NBRC 16637 = DSM 17717	GCA_000621265.1	4.77	61.7	48	4282	4178	Scaffold
P. fuscovaginae CB98818	GCA_000280575.1	6.54	61.4	261	5981	5025	Contig
P. fuscovaginae DAR 77795	GCA_000467005.1	6.25	61.3	482	5872	4347	Contig
P. fuscovaginae DAR 77800	GCA_000467025.1	5.97	61.1	791	-	-	Contig
P. fuscovaginae ICMP 5940	GCA_000467065.1	6.38	61.2	459	6043	4360	Contig
P. fuscovaginae SE-1	GCA_000364705.1	6.53	63.1	407	5868	5786	Contig
P. fuscovaginae UPB0736	GCA_000251185.1	6.71	61.5	102	5664	5585	Scaffold
P. luteola XLDN4-9	GCA_000282775.1	4.63	54.2	231	4402	4299	Contig
P. mandelii 36MFCvi1.1[a]	GCA_000381285.1	6.55	59.2	29	5898	5801	Scaffold
P. mandelii JR-1[a]	GCA_000257545.3	7.19	59.0	2	6560	6420	Complete Genome
P. mendocina DLHK	GCA_000287395.1	5.07	64.7	33	4677	4621	Contig
P. mendocina EGD-AQ5	GCA_000465575.1	5.23	62.7	19	4845	4762	Contig
P. mendocina NK-01	GCA_000204295.1	5.43	62.5	1	5045	4951	Complete Genome
P. mendocina S5.2	GCA_000733715.1	5.40	62.4	3	5094	4964	Contig
P. mendocina ymp	GCA_000016565.1	5.07	64.7	1	4661	4564	Complete Genome
P. monteilii NBRC 103158	GCA_000621245.1	6.31	61.5	85	5944	5815	Scaffold

P. monteilii SB3078	GCA_000510285.1	6.00	62.5	1	5464	5339	Complete Genome
P. monteilii SB3101	GCA_000510325.1	5.95	62.5	1	5393	5267	Complete Genome
P. nitroreducens HBP1	GCA_000518065.1	7.41	64.2	212	7071	6348	Contig
P. nitroreducens TX1	GCA_000313755.1	6.69	64.5	138	6230	6160	Contig
P. oleovorans MOIL14HWK12	GCA_000510765.1	5.01	66.3	12	4587	4519	Scaffold
P. otitidis LNU-E-001	GCA_000632755.1	6.30	66.4	69	5816	5738	Scaffold
P. pelagia CL-AP6	GCA_000410875.1	4.64	57.4	81	4223	4136	Contig
P. plecoglossicida NB2011	GCA_000412715.1	5.41	62.8	85	4952	4883	Scaffold
P. plecoglossicida NBRC 103162	GCA_000688275.1	5.35	63.0	58	4881	4745	Contig
P. poae RE*1-1-14[a]	GCA_000336465.1	5.51	60.8	1	4826	4718	Complete Genome
P. protegens CHA0[a]	GCA_000397205.1	6.87	63.4	1	6176	6080	Complete Genome
P. protegens Pf-5[a]	GCA_000012265.1	7.07	63.3	1	6318	6197	Complete Genome
P. pseudoalcaligenes KF707	GCA_000262065.3	6.68	65.4	229	6211	6111	Contig
P. psychrotolerans L19	GCA_000236825.2	5.10	65.7	53	4819	4759	Contig
P. putida B6-2	GCA_000226035.2	6.24	61.6	27	5685	5511	Contig
P. putida BIRD-1	GCA_000183645.1	5.73	61.7	1	5169	5034	Complete Genome
P. putida CSV86	GCA_000319305.1	6.47	63.1	209	5900	5836	Contig
P. putida DOT-T1E	GCA_000281215.1	6.26	61.4	1	5803	5721	Complete Genome
P. putida F1	GCA_000016865.1	5.96	61.9	1	5341	5220	Complete Genome
P. putida GB-1	GCA_000019125.1	6.08	61.9	1	5493	5369	Complete Genome
P. putida H8234	GCA_000410575.1	6.87	61.6	1	6366	6204	Complete Genome
P. putida HB3267	GCA_000325725.1	5.96	62.6	2	5443	5322	Complete Genome
P. putida KT2440	GCA_000007565.1	6.18	61.5	1	5516	5350	Complete Genome
P. putida LS46	GCA_000294445.2	5.87	61.7	32	5346	5248	Contig
P. putida MTCC 5279	GCA_000411615.1	5.22	62.5	171	4836	4674	Contig
P. putida NBRC 14164	GCA_000412675.1	6.16	62.3	1	5489	5363	Complete Genome
P. putida ND6	GCA_000264665.1	6.20	61.7	2	5593	5454	Complete Genome
P. putida OUS82	GCA_000507325.1	6.63	61.8	164	6172	6025	Contig
P. putida S11	GCA_000292775.1	5.97	62.4	196	3683	3599	Contig
P. putida S13.1.2	GCA_000498395.2	6.72	62.3	15	5952	5694	Contig
P. putida S16	GCA_000219705.1	5.98	62.3	1	5442	5268	Complete Genome
P. putida S610	GCA_000497385.1	4.60	62.0	69	4187	4126	Contig
P. putida SJ3	GCA_000478865.1	6.33	61.7	424	5945	5271	Contig
P. putida SJTE-1	GCA_000271965.1	5.55	62.3	207	4984	4859	Contig
P. putida T2-2	GCA_000710785.1	5.52	62.6	389	5197	5091	Contig
P. putida TRO1	GCA_000367825.1	6.32	61.4	222	5905	5840	Contig
P. putida W619	GCA_000019445.1	5.77	61.4	1	5288	5166	Complete Genome
P. resinovorans NBRC 106553	GCA_000412695.1	6.48	65.3	2	5832	5723	Complete Genome
P. savastanoi pv. *savastanoi* NCPPB 3335	GCA_000164015.2	5.87	57.9	112	5035	4841	Chromosome
P. sp. 2-92(2010)[a]	GCA_000503215.1	6.42	60.4	19	5873	5753	Scaffold
P. sp. 35MFCvi1.1[a]	GCA_000378525.1	6.46	59.4	26	5846	5765	Scaffold
P. sp. 45MFCol3.1[a]	GCA_000382025.1	6.56	59.4	21	5882	5797	Scaffold
P. sp. Ag1[a]	GCA_000278565.1	7.25	60.5	113	6623	6570	Contig
P. sp. CBZ-4[a]	GCA_000346755.1	6.50	61.2	186	5803	5701	Contig
P. sp. CF150[a]	GCA_000416175.1	6.09	59.8	95	5598	5539	Contig
P. sp. CFII64	GCA_000416235.1	6.42	58.9	54	5895	5838	Contig
P. sp. CFII68[a]	GCA_000416195.1	5.92	60.8	128	5378	5323	Contig

P. sp. CFT9[a]	GCA_000416255.1	6.21	59.8	67	5795	5733	Contig
P. sp. CHM02[a]	GCA_000612585.1	6.66	60.9	132	6093	5990	Contig
P. sp. Chol1	GCA_000306015.1	4.87	64.0	199	4578	4508	Contig
P. sp. CMAA1215[a]	GCA_000474765.1	6.66	63.8	224	6380	6111	Contig
P. sp. EGD-AK9[a]	GCA_000465935.1	5.11	65.6	1016	5053	4974	Contig
P. sp. FH1[a]	GCA_000510895.1	7.07	60.1	147	6631	6572	Contig
P. sp. FH4[a]	GCA_000510915.1	6.03	60.1	64	5532	5472	Contig
P. sp. G5(2012)[a]	GCA_000408945.1	7.20	59.3	223	6828	6767	Contig
P. sp. GM102[a]	GCA_000282555.1	6.66	59.0	159	6074	6001	Contig
P. sp. GM16[a]	GCA_000282155.1	6.55	59.1	128	5946	5867	Contig
P. sp. GM17[a]	GCA_000282175.1	6.79	62.8	280	6157	6085	Contig
P. sp. GM18[a]	GCA_000282195.1	6.30	59.5	140	5757	5682	Contig
P. sp. GM21[a]	GCA_000282215.1	6.61	58.5	210	6098	6031	Contig
P. sp. GM24[a]	GCA_000282235.1	6.52	59.1	399	5901	5828	Contig
P. sp. GM25[a]	GCA_000282255.1	6.35	60.9	91	5748	5684	Contig
P. sp. GM30[a]	GCA_000282275.2	6.15	60.3	32	5511	5511	Contig
P. sp. GM33[a]	GCA_000282295.1	6.73	60.1	205	6123	6061	Contig
P. sp. GM41(2012)[a]	GCA_000282315.2	6.69	59.0	13	5975	5975	Contig
P. sp. GM48[a]	GCA_000282335.1	6.44	59.4	200	5906	5845	Contig
P. sp. GM49[a]	GCA_000282355.1	6.59	59.6	345	6300	6227	Contig
P. sp. GM50[a]	GCA_000282375.1	6.69	59.0	155	6104	6033	Contig
P. sp. GM55[a]	GCA_000282395.1	6.49	59.7	163	6008	5949	Contig
P. sp. GM60[a]	GCA_000282415.1	6.42	59.6	181	5943	5884	Contig
P. sp. GM67[a]	GCA_000282435.1	6.50	59.6	183	6027	5966	Contig
P. sp. GM74[a]	GCA_000282455.1	6.10	60.1	180	5592	5531	Contig
P. sp. GM78[a]	GCA_000282475.1	7.29	60.2	235	6741	6681	Contig
P. sp. GM79[a]	GCA_000282495.1	6.71	58.8	126	6087	6026	Contig
P. sp. GM80[a]	GCA_000282515.1	6.79	59.2	282	6247	6183	Contig
P. sp. GM84[a]	GCA_000282535.1	5.82	63.2	384	5316	5255	Contig
P. sp. H1h[a]	GCA_000633255.1	6.37	60.3	78	5690	5592	Contig
P. sp. HPB0071	GCA_000478505.2	5.70	55.2	9	5415	5337	Scaffold
P. sp. HYS	GCA_000259195.1	5.65	62.4	64	5172	5057	Scaffold
P. sp. LAIL14HWK12:I12	GCA_000514335.1	5.58	62.2	26	5048	4958	Scaffold
P. sp. M1	GCA_000317185.3	6.98	67.3	4	6163	6023	Contig
P. sp. M47T1	GCA_000263855.1	6.31	62.5	88	5753	5678	Contig
P. sp. P179	GCA_000478485.2	6.89	65.8	13	6363	6293	Scaffold
P. sp. P818	GCA_000418555.1	5.09	63.4	33	4713	4599	Contig
P. sp. PAMC 25886[a]	GCA_000242655.2	7.02	61.2	95	6368	6276	Contig
P. sp. PAMC 26793[a]	GCA_000313235.1	6.77	60.6	58	6194	6077	Contig
P. sp. PH1b[a]	GCA_000633395.1	7.43	62.9	89	6674	6553	Contig
P. sp. QTF5[a]	GCA_000512695.1	6.04	58.7	110	5513	5370	Contig
P. sp. R62[a]	GCA_000257605.1	6.58	60.0	192	5762	5703	Scaffold
P. sp. R81[a]	GCA_000257625.1	6.23	60.5	8	5699	5599	Scaffold
P. sp. RL	GCA_000647775.1	3.81	65.6	228	3618	3559	Contig
P. sp. S13.1.2	GCA_000292285.1	4.96	62.0	337	4619	4540	Contig
P. sp. TJI-51	GCA_000190455.2	5.72	62.1	1069	6109	6036	Contig
P. sp. TKP[a]	GCA_000508205.1	7.01	60.5	1	6267	6152	Complete Genome

P. sp. UK4[a]	GCA_000174915.1	5.76	60.4	2971	7	6	Contig
P. sp. URIL14HWK12:I6[a]	GCA_000514195.1	6.34	60.0	61	5566	5490	Scaffold
P. sp. URIL14HWK12:I7[a]	GCA_000514275.1	6.88	60.7	78	6095	6016	Scaffold
P. sp. URMO17WK12:I12[a]	GCA_000514395.1	6.57	59.1	32	5996	5897	Scaffold
P. sp. UW4[a]	GCA_000316175.1	6.18	60.1	1	5516	5393	Complete Genome
P. sp. VLB120	GCA_000494915.1	5.97	61.5	2	5539	5412	Complete Genome
P. stutzeri A1501	GCA_000013785.1	4.57	63.9	1	4199	4093	Complete Genome
P. stutzeri ATCC 14405	GCA_000237885.2	4.53	61.4	130	4181	4126	Contig
P. stutzeri ATCC 17588	GCA_000219605.1	4.55	63.9	1	4181	4051	Complete Genome
P. stutzeri B1SMN1	GCA_000416345.1	5.32	63.4	78	5161	5103	Contig
P. stutzeri CCUG 29243	GCA_000267545.1	4.71	62.7	1	4361	4237	Complete Genome
P. stutzeri DSM 10701	GCA_000279165.1	4.17	63.2	1	3875	3765	Complete Genome
P. stutzeri DSM 4166	GCA_000195105.1	4.69	64.0	1	4357	4238	Complete Genome
P. stutzeri KOS6	GCA_000307775.2	4.95	62.9	5	4557	4464	Scaffold
P. stutzeri NF13	GCA_000341615.1	4.67	63.0	82	4379	4321	Contig
P. stutzeri RCH2	GCA_000327065.1	4.60	62.5	4	4306	4205	Complete Genome
P. stutzeri SDM-LAC	GCA_000235745.2	4.23	60.5	199	3928	3840	Contig
P. stutzeri T13	GCA_000282955.1	4.65	63.9	71	4410	4325	Contig
P. stutzeri TS44	GCA_000263395.1	4.28	64.4	78	4060	4000	Contig
P. stutzeri XLDN-R	GCA_000280555.1	4.70	63.9	167	4414	4313	Contig
P. synxantha BG33R[a]	GCA_000263715.2	6.30	59.7	1	5580	5445	Chromosome
P. syringae BRIP34876	GCA_000334035.1	6.02	58.9	99	5157	5098	Scaffold
P. syringae BRIP34881	GCA_000334055.1	6.02	58.9	96	5161	5101	Scaffold
P. syringae BRIP39023	GCA_000333995.1	5.94	59.2	34	5174	5112	Scaffold
P. syringae CC1416	GCA_000452845.2	5.92	59.0	336	5254	5135	Contig
P. syringae CC1417	GCA_000452825.2	5.65	59.3	210	5079	4999	Contig
P. syringae CC1458	GCA_000452805.2	5.80	59.1	364	5158	5049	Contig
P. syringae CC1466	GCA_000452785.2	5.59	58.5	294	5096	4984	Contig
P. syringae CC1513	GCA_000452765.2	5.73	57.9	164	5218	5112	Contig
P. syringae CC1524	GCA_000452745.2	5.83	59.1	264	5261	5181	Contig
P. syringae CC1543	GCA_000452725.2	5.78	59.2	441	5262	5148	Contig
P. syringae CC1544	GCA_000452905.2	5.88	59.1	376	5263	5170	Contig
P. syringae CC1559	GCA_000452685.2	5.86	58.9	365	5245	5152	Contig
P. syringae CC1583	GCA_000452665.2	5.51	59.0	308	4943	4849	Contig
P. syringae CC1629	GCA_000452645.2	5.93	57.7	261	5500	5344	Contig
P. syringae CC1630	GCA_000452625.2	6.06	58.5	283	5473	5361	Contig
P. syringae CC440	GCA_000452605.2	5.73	59.1	354	5108	5011	Contig
P. syringae CC457	GCA_000452585.2	5.84	59.1	356	5212	5106	Contig
P. syringae CC94	GCA_000452925.2	6.15	59.3	566	5578	5455	Contig
P. syringae DSM 10604	GCA_000597765.1	6.07	59.0	84	5238	5120	Contig
P. syringae ICMP 18806	GCA_000344415.1	6.26	58.7	284	5538	5448	Contig
P. syringae KCTC 12500	GCA_000507185.1	6.10	59.0	18	5226	5100	Scaffold
P. syringae pv. *actinidiae* CFBP 7286	GCA_000245415.1	6.14	58.5	351	5539	5415	Scaffold
P. syringae pv. *actinidiae* CH2010-6	GCA_000245475.1	6.20	58.5	342	5639	5504	Scaffold
P. syringae pv. *actinidiae* CRAFRU8.43	GCA_000233815.2	6.14	58.5	585	5566	5432	Contig
P. syringae pv. *actinidiae* ICMP 18708	GCA_000344355.1	6.23	58.5	445	5646	5506	Contig
P. syringae pv. *actinidiae* ICMP 18744	GCA_000342185.1	6.24	58.5	442	5628	5487	Contig

P. syringae pv. *actinidiae* ICMP 18800	GCA_000344375.1	6.23	58.5	463	5635	5497	Contig
P. syringae pv. *actinidiae* ICMP 18801	GCA_000416945.1	6.16	58.6	631	5632	5583	Contig
P. syringae pv. *actinidiae* ICMP 18804	GCA_000344395.1	6.25	58.7	263	5513	5425	Contig
P. syringae pv. *actinidiae* ICMP 18807	GCA_000344535.1	6.13	58.8	148	5403	5288	Contig
P. syringae pv. *actinidiae* ICMP 18883	GCA_000416785.1	6.31	58.7	416	5706	5646	Contig
P. syringae pv. *actinidiae* ICMP 18886	GCA_000416925.1	6.03	58.4	1755	6575	6520	Contig
P. syringae pv. *actinidiae* ICMP 19068	GCA_000416705.1	6.10	58.7	647	5634	5576	Contig
P. syringae pv. *actinidiae* ICMP 19071	GCA_000416485.1	6.02	58.8	391	5511	5448	Contig
P. syringae pv. *actinidiae* ICMP 19072	GCA_000416885.1	6.01	58.8	290	5452	5388	Contig
P. syringae pv. *actinidiae* ICMP 19073	GCA_000416505.1	5.97	58.8	745	5609	5563	Contig
P. syringae pv. *actinidiae* ICMP 19094	GCA_000416745.1	6.29	58.7	497	5711	5653	Contig
P. syringae pv. *actinidiae* ICMP 19095	GCA_000416765.1	6.20	58.7	739	5801	5747	Contig
P. syringae pv. *actinidiae* ICMP 19097	GCA_000416725.1	6.17	58.6	676	5681	5629	Contig
P. syringae pv. *actinidiae* ICMP 19098	GCA_000416545.1	6.31	58.7	315	5650	5592	Contig
P. syringae pv. *actinidiae* ICMP 19099	GCA_000416805.1	6.23	58.7	342	5572	5514	Contig
P. syringae pv. *actinidiae* ICMP 19100	GCA_000416825.1	6.22	58.7	533	5630	5572	Contig
P. syringae pv. *actinidiae* ICMP 19101	GCA_000416585.1	6.00	58.4	1851	6632	6584	Contig
P. syringae pv. *actinidiae* ICMP 19102	GCA_000416845.1	6.01	58.8	468	5425	5369	Contig
P. syringae pv. *actinidiae* ICMP 19103	GCA_000416525.1	6.01	58.8	444	5422	5363	Contig
P. syringae pv. *actinidiae* ICMP 19104	GCA_000416865.1	5.96	58.8	434	5387	5326	Contig
P. syringae pv. *actinidiae* ICMP 19439	GCA_000344555.1	6.22	58.5	477	5647	5509	Contig
P. syringae pv. *actinidiae* ICMP 19455	GCA_000344515.1	6.23	58.5	415	5670	5530	Contig
P. syringae pv. *actinidiae* ICMP 9853	GCA_000344335.1	6.00	58.8	515	5467	5343	Contig
P. syringae pv. *actinidiae* ICMP 9855	GCA_000416665.1	5.93	58.8	375	5327	5265	Contig
P. syringae pv. *actinidiae* M302091	GCA_000145865.1	5.90	58.8	414	5669	5626	Scaffold
P. syringae pv. *actinidiae* NCPPB 3739	GCA_000233835.2	5.93	58.8	815	5558	5445	Contig
P. syringae pv. *actinidiae* NCPPB 3871	GCA_000233795.2	5.94	58.8	462	5351	5238	Contig
P. syringae pv. *actinidiae* PA459	GCA_000245455.1	6.46	58.4	390	6015	5892	Scaffold
P. syringae pv. *actinidiae* Shaanxi M228	GCA_000344475.2	6.35	58.4	419	5763	5604	Contig
P. syringae pv. *actinidiae* TP1	GCA_000344435.1	6.22	58.5	472	5649	5507	Contig
P. syringae pv. *actinidiae* TP6-1	GCA_000344455.1	6.22	58.5	483	5639	5500	Contig
P. syringae pv. *aesculi* 089323	GCA_000145685.1	6.01	58.3	463	5767	5727	Scaffold
P. syringae pv. *aesculi* 2250	GCA_000163275.1	5.99	58.3	364	5465	5254	Scaffold
P. syringae pv. *aesculi* NCPPB 3681	GCA_000163255.1	5.90	58.3	557	5534	5370	Scaffold
P. syringae pv. *aptata* DSM 50252	GCA_000145905.1	6.36	59.1	2562	6408	6368	Scaffold
P. syringae pv. *atrofaciens* DSM 50255	GCA_000498595.1	5.78	59.2	669	5370	5301	Contig
P. syringae pv. *atrofaciens* LMG 5095	GCA_000710085.1	5.89	58.9	1007	5801	5575	Contig
P. syringae pv. *avellanae* ISPaVe013	GCA_000302795.1	6.06	59.1	37	4714	4642	Scaffold
P. syringae pv. *avellanae* ISPaVe037	GCA_000302815.1	5.88	59.2	39	4808	4755	Scaffold
P. syringae pv. *glycinea* B076	GCA_000187045.2	6.24	57.8	104	5660	5580	Contig
P. syringae pv. *glycinea* race 4	GCA_000143005.1	6.23	58.0	3012	8076	8035	Scaffold
P. syringae pv. *lachrymans* M301315	GCA_000146005.1	6.80	56.9	372	7841	7721	Scaffold
P. syringae pv. *lachrymans* M302278	GCA_000145885.1	5.94	58.6	302	5670	5636	Scaffold
P. syringae pv. *mori* 301020	GCA_000145765.1	6.51	57.8	2281	7595	7544	Scaffold
P. syringae pv. *morsprunorum* M302280	GCA_000145745.1	6.09	58.6	445	5880	5837	Scaffold
P. syringae pv. *oryzae* 16	GCA_000156995.1	6.78	57.9	2109	7933	7837	Scaffold
P. syringae pv. *panici* LMG 2367	GCA_000282735.1	5.99	59.0	148	-	-	Contig

P. syringae pv. *phaseolicola* 1448A	GCA_000012205.1	6.11	57.9	3	5493	5233	Complete Genome
P. syringae pv. *pisi* PP1	GCA_000452445.2	5.95	58.8	256	5311	5200	Contig
P. syringae pv. *syringae* 1212	GCA_000452465.2	6.16	59.1	338	5432	5320	Contig
P. syringae pv. *syringae* 642	GCA_000177515.1	5.81	59.2	296	5202	5126	Contig
P. syringae pv. *syringae* B301D-R	GCA_000585725.1	6.04	59.2	81	5273	5185	Contig
P. syringae pv. *syringae* B64	GCA_000331385.1	5.93	59.0	1	5055	4944	Chromosome
P. syringae pv. *syringae* B728a	GCA_000012245.1	6.09	59.2	1	5220	5089	Complete Genome
P. syringae pv. *syringae* SM	GCA_000412165.1	6.12	59.0	2	5325	5181	Chromosome
P. syringae pv. *tabaci* 6605	GCA_000275945.1	6.16	58.0	283	5416	5142	Contig
P. syringae pv. *tabaci* ATCC 11528	GCA_000145945.1	6.21	58.1	1064	6347	6299	Scaffold
P. syringae pv. *theae* ICMP 3923	GCA_000416465.1	6.26	58.6	378	5697	5644	Contig
P. syringae pv. *theae* NCPPB 2598	GCA_000245395.1	6.65	58.5	216	6511	6393	Scaffold
P. syringae pv. *tomato* DC3000	GCA_000007805.1	6.54	58.3	3	5842	5619	Complete Genome
P. syringae pv. *tomato* K40	GCA_000177455.1	6.15	58.6	582	5730	5625	Contig
P. syringae pv. *tomato* T1	GCA_000172895.1	6.15	58.6	122	5783	5702	Contig
P. syringae UB246	GCA_000452865.1	6.27	57.1	410	5254	5207	Scaffold
P. syringae UB303	GCA_000452565.2	5.99	59.2	478	5424	5288	Contig
P. syringae USA007	GCA_000452545.2	5.97	58.9	369	5317	5195	Contig
P. syringae USA011	GCA_000452525.2	6.43	59.2	198	5503	5399	Contig
P. taeanensis MS-3	GCA_000498575.1	5.46	60.9	19	4705	4594	Contig
P. taiwanensis SJ9	GCA_000500605.1	6.25	61.8	736	6093	5519	Contig
P. thermotolerans J53	GCA_000513835.1	3.75	67.0	62	3529	3435	Scaffold
P. tolaasii 6264[a]	GCA_000316215.1	6.23	61.0	688	5970	5791	Contig
P. tolaasii PMS117[a]	GCA_000276565.1	7.01	60.3	357	6355	6128	Contig
P. umsongensis 20MFCvi1.1[a]	GCA_000377725.1	6.46	59.4	27	5838	5761	Scaffold
P. umsongensis UNC430CL58Col[a]	GCA_000620285.1	6.46	59.3	28	5835	5748	Scaffold
P. veronii 1YdBTEX2[a]	GCA_000350565.1	6.68	60.6	63	6012	5901	Contig
P. viridiflava CC1582	GCA_000452505.1	6.02	59.3	211	5400	5333	Scaffold
P. viridiflava TA043	GCA_000452485.1	5.98	59.3	218	5305	5240	Scaffold
P. viridiflava UASWS0038	GCA_000307715.1	5.91	59.3	201	5299	5233	Contig

[a] Genomes belonging to the *P. fluorescens* complex.
