# Supplementary figures and images for: Correction: Genomic and Genetic Diversity within the Pseudomonas fluorescens Complex
Source: PLoS One. 2016 Apr 11;11(4):e0153733. doi: 10.1371/journal.pone.0153733 (PMC4827820; doi:10.1371/journal.pone.0153733)

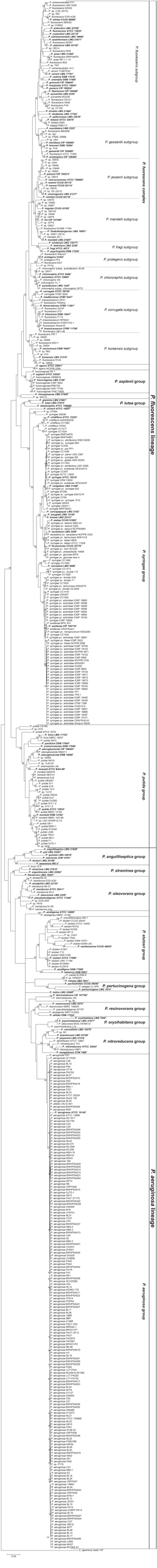

Supplement: S1 Fig — MLSA based on partial sequences of 16S rDNA, gyrB, rpoD and rpoB genes from 451 sequenced genomes and 107 type strains (bold), ML method and Tamura-Nei model. C. japonicus Ueda 107 was used as outgroup. Only bootstrap values above 75% over 1000 replicates are shown. Bold and T indicates type strain. (PDF) [file pone.0153733.s001.pdf]
